# Early Prediction and Prognosis in Acute Kidney Injury Using a Biomarker-Aware Transformer: Retrospective Multicenter Study

**DOI:** 10.2196/90687

**Published:** 2026-10-26

**Authors:** Zijian Xu, Deyi Li, Qi Xu, Alan SL Yu, Elizabeth A Chrischilles, Lindsay G Cowell, Kathleen M McTigue, Raghavan Murugan, Dana Y Fuhrman, Mei Liu

**Affiliations:** 1 Department of Health Outcomes and Biomedical Informatics College of Medicine University of Florida Gainesville, FL United States; 2 Division of Nephrology and Hypertension and the Kidney Institute University of Kansas Medical Center Kansas City, KS United States; 3 Department of Epidemiology College of Public Health University of Iowa Iowa City, IA United States; 4 Department of Health Data Science and Biostatistics Peter O’Donnell Jr. School of Public Health The University of Texas Southwestern Medical Center Dallas, TX United States; 5 Department of Medicine School of Medicine University of Pittsburgh Pittsburgh, PA United States; 6 Department of Critical Care Medicine, Program for Critical Care Nephrology School of Medicine University of Pittsburgh Pittsburgh, PA United States; 7 Divisions of Critical Care Medicine and Nephrology Cincinnati Children's Hospital Medical Center Cincinnati, OH United States

**Keywords:** acute kidney injury, clinical risk prediction, deep learning, EHR, electronic health record, multisite validation, transformer models

## Abstract

**Background:**

Acute kidney injury (AKI) is a common, life-threatening complication among hospitalized patients. Timely prediction is crucial for prevention, while accurate prognosis following onset is essential for guiding care. Although electronic health records (EHRs) provide rich longitudinal data, their high dimensionality, heterogeneity, and temporal complexity pose challenges for traditional machine learning.

**Objective:**

The aim of this study was to develop and evaluate a transformer-based model for generalizable AKI prediction and prognosis by modeling long-range temporal dependencies and complex relationships in heterogeneous EHR data and to examine its interpretability.

**Methods:**

We developed the Biomarker-Aware Transformer for AKI (BAT-AKI), which integrates multidimensional embeddings capturing temporal dynamics, textual descriptions, hierarchical ontology structures, and semantic relationships in EHRs. To enhance sensitivity to AKI-related biomarker fluctuations, BAT-AKI combines masked language modeling with a novel biomarker abnormality-aware pretraining task. The model was pretrained and fine-tuned for 4 tasks: AKI onset, early reversal, recovery at discharge, and 90-day post-AKI mortality. Pretraining and internal validation used data from the National Patient-Centered Clinical Research Network (PCORnet) site, with external validation across 3 additional sites.

**Results:**

BAT-AKI achieved the strongest overall discrimination, outperforming baseline models in area under the receiver operating characteristic curve (AUROC) and area under the precision-recall curve (AUPRC) on most tasks. For AKI onset prediction, it achieved an AUROC of 0.8549 (95% CI 0.8433-0.8658) and an AUPRC of 0.6041 (95% CI 0.5786-0.6292), exceeding the second-best baseline by 2.8% in AUROC and 8.1% in AUPRC. Across prognostic tasks, BAT-AKI achieved AUROCs of 0.6006 (95% CI 0.5703-0.6318), 0.6080 (95% CI 0.5745-0.6397), and 0.8017 (95% CI 0.7708-0.8311) and AUPRCs of 0.7036 (95% CI 0.6690-0.7388), 0.7417 (95% CI 0.7082-0.7765), and 0.5063 (95% CI 0.4422-0.5684) for early reversal, recovery at discharge, and 90-day post-AKI mortality, respectively, corresponding to relative gains of 2.4% to 6% in AUROC and 3.7% to 4.1% in AUPRC over the second-best baseline. Performance remained stable across external cohorts, with AKI onset AUROCs ranging from 0.7754 (95% CI 0.7677-0.7827) to 0.7989 (95% CI 0.7885-0.8092) and AUPRCs ranging from 0.4777 (95% CI 0.4631-0.4924) to 0.5687 (95% CI 0.5495-0.5871), corresponding to relative improvements of 3.7% to 5.3% in AUROC and 16.8% to 25.3% in AUPRC over the transformer baseline. In a low-resource setting using only 2% of the fine-tuning data, BAT-AKI achieved an AUROC of 0.7898 (95% CI 0.7759-0.8036) and an AUPRC of 0.4903 (95% CI 0.4640-0.5178) for AKI onset prediction, exceeding the second-best baseline by 4.2% in AUROC and 10.9% in AUPRC. Visualization of embeddings, attention patterns, and integrated-gradient feature attributions highlighted clinically meaningful relationships and patient-specific trajectories.

**Conclusions:**

BAT-AKI provides generalizable AKI prediction and prognosis with consistent discrimination across institutions, while offering interpretability insights into its predictions, supporting its potential for early warning and AKI care optimization.

## Introduction

Acute kidney injury (AKI) is a frequent and serious complication of hospitalization, affecting approximately 1 in 5 adult inpatients and nearly half of patients admitted to intensive care units [1]. AKI is associated with substantial short- and long-term morbidity and mortality, including in-hospital mortality rates of 10% to 20%, progression to chronic kidney disease (CKD) in up to 25% of survivors within 3 years, and increased risk of recurrent or persistent kidney dysfunction and cardiovascular complications after discharge [1,2]. Given its high prevalence and severe clinical consequences, timely prediction of AKI is essential for prevention, while accurate prognosis after AKI onset is critical for guiding post-AKI management and mitigating downstream morbidity [3].

The widespread implementation of electronic health record (EHR) systems has enabled new opportunities for data-driven AKI risk stratification [4]. EHRs capture rich, longitudinal, and multidimensional information on patients—including demographics, vital signs, diagnoses, procedures, laboratory measurements, and medications—providing a detailed view of each patient’s evolving clinical trajectory that offers a solid foundation for personalized AKI prediction and prognostic modeling [5].

However, most existing AKI prediction studies rely on traditional machine learning methods that represent EHR data as cross-sectional features aggregated over fixed time windows [6-12]. Such representations obscure temporal dependencies and dynamic biomarker patterns that are central to AKI pathophysiology. To better model longitudinal data, deep learning models have increasingly been applied. Recurrent neural networks, including long short-term memory networks and gated recurrent units, have shown improved performance for AKI-related prediction tasks [13-15] but struggle to capture long-range dependencies in heterogeneous patient trajectories and provide limited token-level (ie, individual clinical events) interpretability, which is important for clinical reasoning and trust.

Transformer-based architectures overcome many of these limitations [16]. Originally developed for natural language processing and later adapted to computer vision, transformers enable flexible modeling of long-range temporal dependencies through self-attention mechanisms and support pretraining on large-scale unlabeled data followed by fine-tuning for task-specific prediction. This paradigm is particularly well suited for EHR data, where labeled outcomes may be limited but longitudinal records are abundant. Recent transformer-based models such as BEHRT [17], G-BERT [18], and DeepJ [19] treat medical codes as tokens in patient sequences, enabling models to learn contextual relationships among clinical events and supporting a range of downstream clinical prediction tasks such as mortality, length of stay, and readmission.

Despite these advances, existing transformer-based clinical prediction models remain limited in several aspects. First, many employ generic transformer architectures with simplified embedding schemes and standard pretraining objectives that insufficiently leverage the structured nature of EHRs and the clinical context of AKI. For example, at the embedding level, each medical code inherently corresponds to a clinically meaningful event with associated textual descriptions and ontology relationships, offering richer semantics than a simple unique identifier. Different code types (eg, diagnoses vs procedures) also exhibit distinct temporal and cooccurrence patterns that reflect underlying disease processes. Second, commonly used pretraining objectives adapted from natural language modeling treat all tokens uniformly and do not explicitly emphasize the temporal dynamics of biomarker changes that are critical for AKI development and progression. Third, most prior studies focus on predicting a single AKI-related outcome (eg, onset or a specific postonset event) in isolation, rather than learning a shared pretrained representation that can be adapted through task-specific fine-tuning to multiple AKI-related outcomes spanning early warning and postonset prognosis, which would support more comprehensive risk assessment for clinical decision-making.

To address these gaps, we propose the Biomarker-Aware Transformer for AKI (BAT-AKI), a transformer-based framework tailored to the clinical characteristics of AKI and pretrained on structured EHR data. Our contributions are threefold:

- Multisource embeddings that integrate medical code types, ontology hierarchies, textual descriptions, and temporal positions of tokens to construct semantically enriched token representations capturing contextual, semantic, and temporal relationships among clinical events.
- Biomarker abnormality-aware pretraining that explicitly models AKI-related token occurrences and biomarker fluctuation patterns, strengthening the model’s ability to learn physiological dynamics preceding onset and characterize AKI progression.
- Comprehensive evaluations across multiple AKI prediction and prognosis tasks, including AKI onset, early reversal, recovery at discharge, and 90-day post-AKI mortality, assessing predictive performance, robustness under limited fine-tuning sample sizes, cross-site generalizability, and interpretability insights derived from embedding visualization, attention analysis, and integrated-gradient attribution.

## Methods

### Overview of the Framework

At a high level, BAT-AKI follows the traditional pretraining-fine-tuning paradigm to model sequences of structured EHR data derived from patients’ longitudinal health care journeys (Figure 1A). The framework aims to capture temporal dependencies and semantic relationships among heterogeneous clinical events to predict various outcomes.

**Figure 1:**
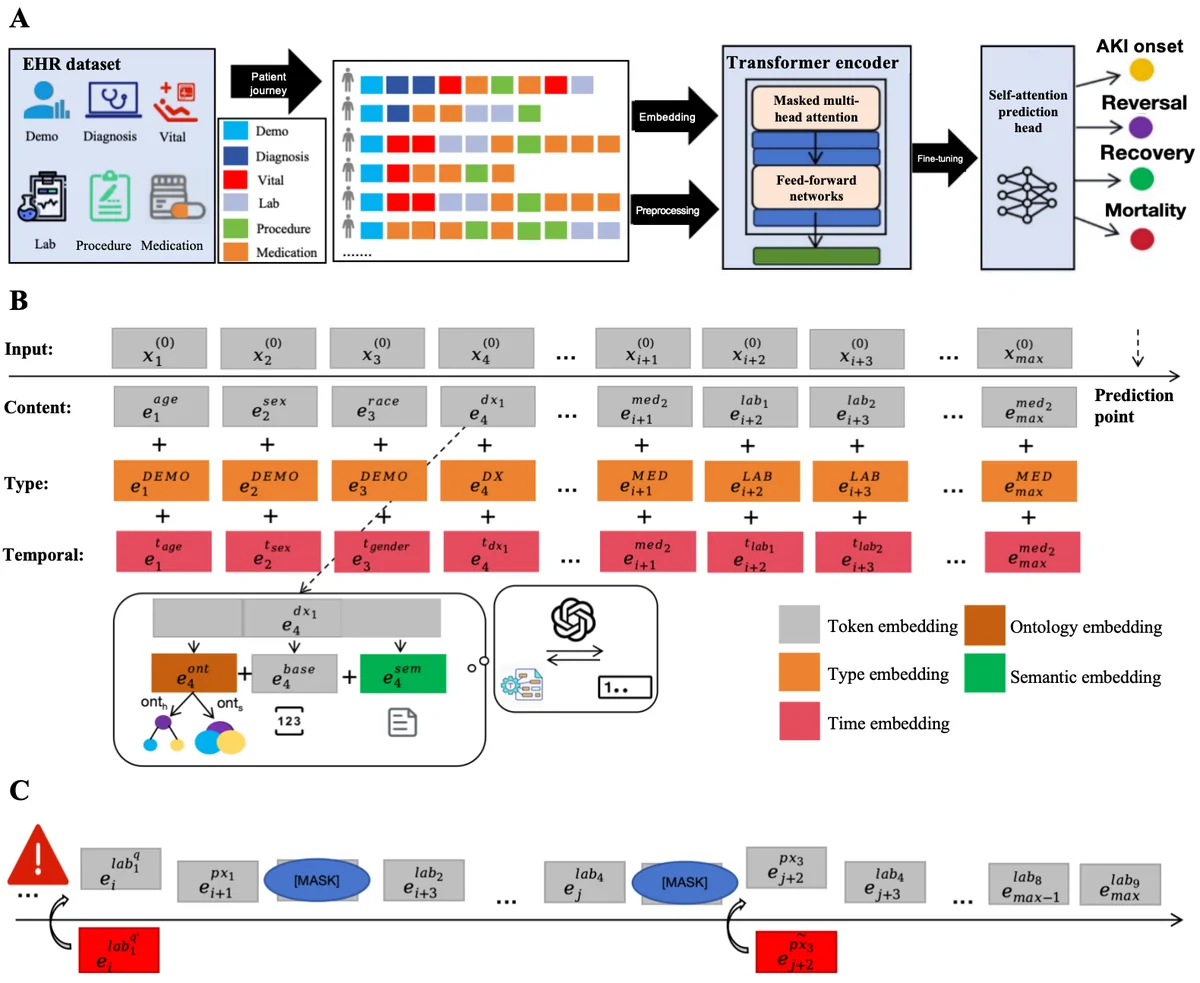
Key components of the Biomarker-Aware Transformer for AKI (BAT-AKI) framework. (A) Overall architecture illustrating patient electronic health record (EHR) sequence construction, transformer-based pretraining, and fine-tuning on 4 prediction tasks. (B) Multisource embedding structure integrating content, type, temporal, ontology, and semantic representations of medical codes to form transformer inputs. (C) Pretraining includes masked language modeling and biomarker abnormality-aware pretraining to learn acute kidney injury (AKI)–related clinical patterns from structured EHR sequences through masking and perturbing tokens, with the red exclamation mark highlighting clinically abnormal biomarker perturbations.

Specifically, BAT-AKI is designed to support both AKI onset prediction and post-AKI prognosis, encompassing the following four prediction tasks: (1) AKI onset, defined as the first occurrence of AKI during hospitalization; (2) AKI early reversal, defined as reversal based on serum creatinine (SCr) falling back below the AKI threshold and remaining stable for at least 24 hours, occurring within 7 days after onset; (3) AKI recovery, defined among patients discharged alive as kidney function recovery at discharge, indicated by the final SCr level returning toward the pre-AKI baseline [20]; and (4) 90-day post-AKI mortality, defined as all-cause death within 90 days following AKI onset. These outcomes were defined according to the Kidney Disease: Improving Global Outcomes criteria [21] and established clinical guidelines [22]. Detailed descriptions are provided in Method S1 in Multimedia Appendix 1.

Building on this design, BAT-AKI comprises three major components corresponding to different stages of the modeling pipeline: (1) multisource embedding—embedding enrichment integrating multisource clinical information to construct semantically rich clinical event sequences (Figure 1B), (2) biomarker abnormality-aware pretraining—extending the standard masked language modeling (MLM) objective with a biomarker abnormality-aware pretraining (BAP) task to explicitly model AKI-related token occurrences and physiological fluctuations (Figure 1C), and (3) model fine-tuning with task-specific prediction heads for downstream evaluation.

### Data Resources and Processing

This study leveraged EHR data from 4 academic health systems within 2 PCORnet clinical research networks—the Greater Plains Collaborative (GPC) and PaTH [23,24]—for model pretraining, internal validation, and external validation. BAT-AKI was pretrained and fine-tuned using University of Kansas Medical Center (KUMC) data from 2009 to 2020 and evaluated on a temporally distinct 2021 KUMC cohort and 3 external PCORnet sites from the same year: the University of Iowa, the University of Texas Southwestern Medical Center, and the University of Pittsburgh.

The study population included adult patients (aged ≥18 years) with at least 2 SCr measurements during hospitalization. To ensure sufficient observation time for AKI development, encounters with a length of stay of less than 2 days were excluded. Additional exclusions were (1) AKI occurring on the day of admission, to focus on hospital-acquired AKI; and (2) patients with prior CKD diagnoses but without available SCr measurements within the preceding year to reliably determine baseline SCr. All eligible encounters were treated as independent visits to maximize data use while ensuring that encounters from the same patient did not appear in both the training and test sets.

We used a unified data-observation window across all downstream tasks. Model inputs included the patient’s complete diagnosis history before the index admission together with clinical events recorded during the index hospitalization. For AKI patients, model inputs comprised clinical events recorded during the index hospitalization through 24 hours before AKI onset; for non-AKI patients, index hospitalization events up to 24 hours before the last SCr measurement were used. Sharing a single preonset window across tasks, rather than tailoring the input to each outcome, enables assessment of how much of the AKI course—onset and the trajectory that follows it—is captured in the representation formed before onset, while producing risk and prognosis together from a single clinically actionable decision point.

Six EHR data categories were used: demographics, diagnoses, procedures, laboratory tests, medications, and vital signs. Data were standardized using *ICD* (*International Classification of Diseases*), Current Procedural Terminology (CPT), Logical Observation Identifiers Names and Codes (LOINC), Anatomical Therapeutic Chemical Classification System (ATC), and RxNorm, with textual, ontological, and semantic information obtained from the coding systems and Unified Medical Language System (UMLS) [25]. Continuous laboratory and vital-sign measurements were discretized into quantile-based categorical tokens. Detailed preprocessing procedures are provided in Method S2 in Multimedia Appendix 1.

### Preliminary on EHR Data

Throughout, we use “clinical event” to refer to a recorded observation (eg, a laboratory measurement, diagnosis, medication, or procedure), “code” to refer to its standardized identifier, and “token” to refer to the discrete unit derived from it that serves as model input. In this study, each hospital patient encounter is represented as a temporally ordered sequence of discrete medical codes corresponding to individual clinical events. Each medical code is defined as a tuple (*c_i_*, *d_i_*, *t_i_*), where *c_i_* ∈ *C*, *di* ∈ *D*, and *t_i_* ∈ *T*. Here, *c_i_* denotes a unique medical code that belongs to the overall code vocabulary *C*; *d_i_* indicates the event type of *D*, belonging to 1 of the 6 categories of EHR data types described above; and *t_i_* is the corresponding timestamp from the event time set *T*. Formally, a patient visit can be expressed as 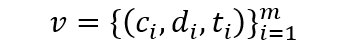, where *m* is the number of events in the sequence, limited by the maximum length *M.* For downstream prediction tasks, each patient visit *v* is associated with a binary labeled outcome vector *y_ft,s_* ∈ {0,1}, where *s* = 1, ... , *S* and *S* is the total number of downstream tasks.

### Embedding Construction

We transform each triplet (*c_i_*, *d_i_*, *t_i_*) into a set of embedding vectors 
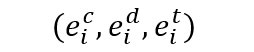
, encoding the code’s content, type, and temporal position. These embeddings are then summed to form the overall code embedding: 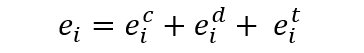 where “+” denotes element-wise vector addition.

First, we initialize a base embedding 
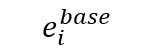
 as a randomly initialized unique identifier for the clinical event *c_i_*. Next, we obtain a semantic embedding 
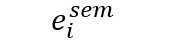
 that captures the textual meaning of the event. Specifically, the official text description of each code is retrieved from the corresponding terminology [26-28] and encoded using a pretrained Sentence-BERT model [29].

We then incorporate hierarchical ontology information by identifying the ancestor concepts of the clinical event *c_i_* within its respective coding hierarchy and obtaining their embeddings, denoted as 
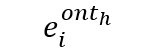
. For example, the *ICD-9* code 250 (diabetes mellitus) belongs to the higher-level category “Endocrine, Nutritional and Metabolic Diseases, and Immunity Disorders,” and its hierarchical ontology embedding is derived accordingly. In parallel, we extract semantic relations from the UMLS by retrieving the semantic type of *c_i_* and encoding it as 
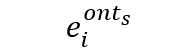
. For instance, the LOINC code 6037-8 (base deficit in blood) is classified in UMLS as “Finding,” a semantic type it shares with diagnostic concepts such as *ICD-9* code 276 (disorders of fluid, electrolyte, and acid-base balance). Unlike the category-based hierarchical structure, the semantic ontology captures cross-terminology relationships that mirror real-world clinical associations. Finally, we define the overall code embedding for the clinical event as the concatenation of these 4 components:

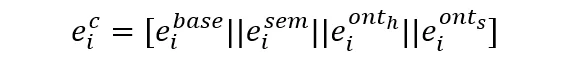



where “||” denotes vector concatenation.

To ensure consistent embedding dimensionality across all tokens, placeholder vectors are assigned when certain subembeddings are unavailable. Further implementation details, including the specific transformations and class definitions used for the semantic and ontology embeddings, are provided in Method S3 in Multimedia Appendix 1.

### Sequence Encoding by Transformer Layers

The resulting embeddings *E* = [*e_1_*,*e_2_*, ... *e_m_*] undergo dropout regularization and are then fed into a transformer encoder [16] as the initial hidden representation *H*^(0)^, which models temporal dependencies and interevent interactions across the entire patient visit sequence. Each encoder layer *l* = 1, ... , *L* consists of a multihead self-attention (MHSA) module [16] followed by a position-wise feed-forward network (FFN), both equipped with residual connections, dropout, and layer normalization (LN):

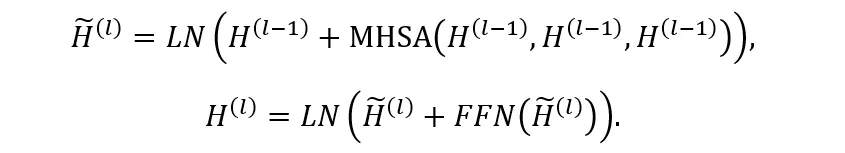



After *L* stacked layers, the encoder outputs contextualized hidden representations:

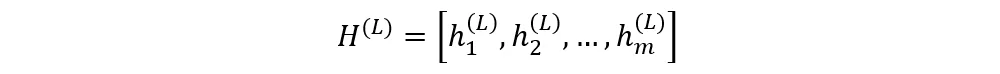



where each hidden state 
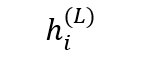
 corresponds to the *i*-th token at the *L*-th layer in the visit sequence and captures its clinical and temporal context by attending to all other tokens within the same encounter.

### Pretraining Objectives

During pretraining, a composite learning objective is adopted. The first component is MLM, following the approach introduced by Devlin et al [30], where a prediction head uses the hidden representations of the unmasked tokens to infer the identity of the masked tokens. Specifically, a proportion *ρ_MLM_* of the tokens in each *v* are randomly masked, resulting in prediction pairs 
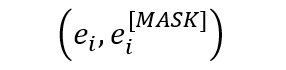
. The loss is defined as:

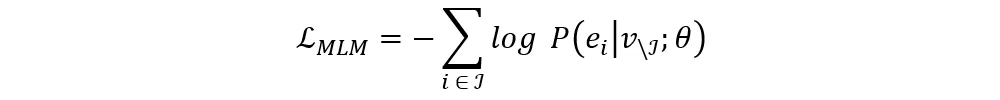



where *I* denotes the set of masked positions in a patient token sequence *v*, *e_i_* is the original token at masked position *I*, *v_\I_* represents the same sequence with tokens in *I* replaced by the special [MASK] token, and *θ* represents the transformer model parameters.

The second component is the BAP objective. Inspired by previous studies leveraging both perturbation-based pretraining [30,31] and replacement-based discriminative objectives [32,33], we propose a novel BAP pretraining objective to enhance the model’s semantic understanding and enable it to capture temporal relationships among different AKI-relevant clinical events. These events were identified by a label-informed selection procedure using encounter-level token-frequency differences with a Mann-Whitney *U* test, then filtered according to prior clinical evidence [12,34,35], forming an AKI-related lexicon *V_aki_* (eg, diagnosis code of “CKD,” laboratory measurement code of “blood urea nitrogen,” and laboratory measurement code of “anion gap”). The detailed composition of *V_aki_* is provided in Method S4 in Multimedia Appendix 1.

For each patient visit sequence *v*, a proportion *ρ_BAP_* of tokens belonging to the AKI-related lexicon *V_aki_* are perturbed according to the following rules:

Each quantitative token *e^q^* ∈ *V_aki_* has a probability *ρ_BAP_* of being perturbed by applying 1 of 3 quantile-based perturbation rules: (1) symmetric inversion: *e^q'^,* where *q^'^* = *Q* + 1 – *q* (eg, when Q=6, quantile 6 ↔ 1, 5 ↔ 2, 4 ↔ 3); (2) random substitution: *q^'^* is randomly sampled from *Q*\{*q*}; (3) cyclic shift: *q'* = ((*q* + 1) mod *Q*) + 1 (eg, 1 → 3, 2 → 4, 3 → 5)

Each categorical token *e^d^* ∈ *V_aki_* with code type *d* ∈ *D*, has a probability *ρ_BAP_* of being perturbed with its replacement *e* ~*^d^*, randomly drawn from the same code-type subset in *V_aki_*.

Each sequence is assigned a binary supervision label vector *y_bap_* = {*y_bap,1_*, *y_bap,2_*, ... , *y_bap,m_*}, where *y_bap,i_* = 1 indicates that the token at timestamp *i* has been perturbed and *y_bap,i_* = 0 otherwise. Accordingly, the BAP loss is formulated as a cross-entropy loss, defined as:

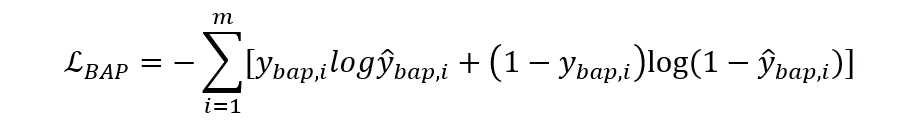



where *y* ^*_bap,i_* denotes the predicted probability, obtained from a prediction head applied to the hidden representation of the token at *i*.

Finally, the overall pretraining objective is defined as a weighted sum of the 2 components:

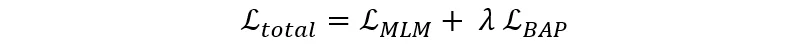



where λ is a weighting coefficient that balances the 2 loss components.

### Patient Representation and Fine-Tuning

After pretraining, each patient sequence *v* is encoded by the transformer encoder into a contextualized hidden representation: *H*^(^*^L^*^)^ = [*h_1_*^(^*^L^*^)^, *h_2_*^(^*^L^*^)^, ... , *h_m_*^(^*^L^*^)^]. These representations can be aggregated by self-attention pooling during the fine-tuning process, which is defined as follows:

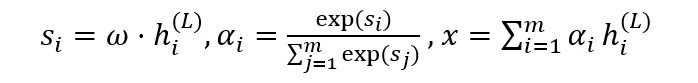



where *ω* is a learnable vector of weights, and *α_i_* are the normalized attention weights assigned to the *i*-th patient token within the patient sequence *v*.

Finally, the patient-level representation *x* is fed into a simple FFN prediction head, *y* ^*_ft,s_* = *FFN*(*x*), to predict each of the *S* downstream outcomes. For the imbalanced AKI onset and 90-day post-AKI mortality tasks, class weighting was applied using *w*_pos_ = *N*_neg_/*N*_pos_, with the negative class weight fixed to 1. Equal class weighting (*w*_pos_ = 1) was used for AKI early reversal and recovery based on validation performance. Model selection and early stopping were based on validation performance, and final performance was evaluated on the test set. *F*_1_-scores were calculated using a fixed probability threshold of .5.

### Model Evaluation

BAT-AKI was evaluated along the following three axes: (1) performance on multiple AKI prediction and prognosis tasks; (2) robustness in low-resource settings by fine-tuning with varying training sizes; and (3) cross-site generalizability in external cohorts. For the primary evaluation, BAT-AKI was compared with 5 baseline models on all 4 downstream tasks using the largest available fine-tuning dataset. To ensure temporal consistency, a fixed 2021 temporal split was used as the test set across all sites. In the low-resource experiments, subsets were drawn from the fixed fine-tuning set by stratified random sampling with respect to the AKI onset label, while the fixed temporal test set was used for evaluation throughout. For each model and task, performance is reported with 95% CIs obtained from 5000 bootstrap resamples of the test set. Models were compared using a paired bootstrap test on the same resamples, with 2-sided *P* values reported [36,37]. We additionally evaluated AKI onset performance within demographic subgroups on the internal temporal test set, stratified by race into White, Black, and other groups and by age into strata of individuals aged younger than 65 years, 65 to 79 years, and 80 years or older, with the transformer baseline as comparator. The other race group included Asian, Native American, and unknown race categories.

We used 5 baseline models. Four are widely benchmarked for AKI and EHR prediction—the vanilla transformer [16], long short-term memory [38], and extreme gradient boosting (XGBoost) [39], and the general-purpose EHR model, Reverse Time Attention (RETAIN) [40]—and the fifth is a recent heterogeneous relation-aware transformer (HEART) [33] pretrained on EHR data. Like BAT-AKI, HEART was pretrained and then fine-tuned on the downstream tasks. The design of the XGBoost baseline is further detailed in Method S5 in Multimedia Appendix 1. All models were evaluated on the same temporal test set using the area under the receiver operating characteristic curve (AUROC), the area under the precision-recall curve (AUPRC), and the *F*_1_-score calculated at a fixed probability threshold of .5.

To evaluate model generalizability, we compared BAT-AKI with a structurally equivalent transformer baseline. Both were developed on the KUMC dataset—BAT-AKI was pretrained and then fine-tuned, whereas the transformer baseline was trained from scratch—and then applied directly to the external sites. Performance was assessed across all 4 downstream prediction tasks.

To ensure fair comparison, all baseline models were implemented under a consistent training framework with aligned input dimensions, optimization strategies, and early stopping criteria while allowing task- and model-specific hyperparameter tuning. Key training configurations, hyperparameter settings, and the computational environment are provided in Table S1 and Method S5 in Multimedia Appendix 1.

Model interpretability was evaluated using 3 approaches. First, t-distributed stochastic neighbor embedding (t-SNE) [41] was applied to visualize static token embeddings in 2D. In parallel, patient-level representations were derived from the demographic-token hidden states of the pretrained BAT-AKI encoder. These representations were reduced with principal component analysis, clustered into subgroups by spectral clustering, and visualized in two dimensions with t-SNE, after which cluster-level AKI incidence was summarized to characterize the subgroups. Second, interpretability was examined through a case study by analyzing token-level attention weights from the transformer encoder to assess temporal patterns in laboratory representations. Third, we applied integrated gradients [42] to quantify feature importance for AKI onset prediction, complementing the case study. Detailed procedures are provided in Method S6 in Multimedia Appendix 1.

### Ethical Considerations

This work constitutes a retrospective secondary analysis of routinely collected clinical records. The institutional review board (IRB) at the University of Florida reviewed the study (IRB202400190) and determined it to be exempt on the basis that it involved only preexisting, deidentified patient records. The records analyzed were generated in the course of routine clinical care, and no participant was recruited, contacted, or observed prospectively for this work. With respect to confidentiality, every record was rendered anonymous under the Safe Harbor provisions of HIPAA (Health Insurance Portability and Accountability Act) before it reached the investigators, and access was granted after review by the GPC and PaTH Data Request Oversight Committee, under executed data use agreements with the GPC and PaTH networks that bar reidentification and prohibit release of record-level data beyond the study team. No compensation arises, as the study entailed no participant contact. Finally, neither the manuscript nor the supplementary materials reproduce any image or other material through which an individual could be recognized.

## Results

### Cohort Description

The study included 247,658 adult encounters from 129,731 unique patients at KUMC (2009-2021), with a mean of 1.9 (SD 2.2) encounters per patient, for model pretraining and internal evaluation along with 3 external validation cohorts from 2021. Within the KUMC cohort, 198,885 encounters were used for pretraining. The fine-tuning cohort comprised 37,909 encounters, including 33,115 training and 4794 validation encounters, while 10,864 encounters constituted the fixed internal temporal test set. The 3 external sites were used entirely as external testing cohorts. In accordance with the applicable data use agreements, the external institutions are reported anonymously as site A, site B, and site C throughout the manuscript. As summarized in Table 1, sex distribution was balanced (male 49.7%-51.9%), mean age ranged from 58.0 (SD 17.3) to 64.7 (SD 17.0) years, baseline SCr values ranged from 0.86 to 0.94 mg/dL, and AKI incidence ranged from 12.9% to 15.6% across sites. Detailed code-based definitions for each comorbidity are provided in Table S2 in Multimedia Appendix 1.

**Table 1:** Demographic, clinical, and comorbidity characteristics of the study cohorts across internal and external sites.

Characteristic				KUMC^a^ (internal)		Site A (external)		Site B (external)		Site C (external)
**Patient characteristic**										
	Patient encounters, n		247,658		15,932		8573		37,295	
	Sex (male), n (%)		126,570 (51.1)		8275 (51.9)		4307 (50.2)		18,530 (49.7)	
	Age (years), mean (SD)		58.0 (17.3)		59.0 (17.6)		60.1 (17.5)		64.7 (17.0)	
	Race (White), n (%)		184,085 (74.3)		14,070 (88.3)		5665 (66.1)		30,967 (83)	
	Race (Black), n (%)		38,901 (15.7)		925 (5.8)		1816 (21.2)		4018 (10.8)	
	**Race (other), n (%)**		24,672 (10)		937 (5.9)		1092 (12.7)		2310 (6.2)	
		Asian	2376 (1)		170 (1.1)		243 (2.8)		248 (0.7)	
		Native American	992 (0.4)		39 (0.2)		30 (0.3)		97 (0.3)	
		Unknown	21,304 (8.6)		728 (4.6)		819 (9.6)		1965 (5.3)	
	Ethnicity (Hispanic or Latino), n (%)		14,517 (5.9)		572 (3.6)		1076 (12.6)		211 (0.6)	
	Ethnicity (not Hispanic or Latino), n (%)		231,081 (93.3)		15,228 (95.6)		7243 (84.5)		33,277 (89.2)	
	Ethnicity (unknown), n (%)		2060 (0.8)		132 (0.8)		254 (3)		3807 (10.2)	
**Clinical outcomes**										
	SCr^b^ baseline (mg/dL), mean (SD)		0.91 (0.34)		0.86 (0.32)		0.90 (0.39)		0.94 (0.40)	
	AKI^c^ incidence, n (%)		31,970 (12.9)		2480 (15.6)		1337 (15.6)		5287 (14.2)	
	AKI early reversal, n (%)^d^		20,254 (63.4)		1647 (66.4)		874 (65.4)		3314 (62.7)	
	AKI recovery, n (%)^e^		19,186 (64.8)		1477 (65.8)		789 (65.5)		2908 (65.1)	
	90-day post-AKI mortality, n (%)^d^		5500 (17.2)		444 (17.9)		263 (19.7)		1294 (24.5)	
**Comorbidities, n (%)**										
	Cancers		57,952 (23.4)		2617 (16.4)		2363 (27.6)		4316 (11.6)	
	CVD^f^		133,631 (54)		7197 (45.2)		5789 (67.5)		24,419 (65.5)	
	CKD^g^		27,028 (10.9)		1465 (9.2)		986 (11.5)		5031 (13.5)	
	Diabetes		47,650 (19.2)		2623 (16.5)		2205 (25.7)		9597 (25.7)	
	Hypertensive disorders		99,307 (40.1)		5292 (33.2)		4224 (49.3)		20,658 (55.4)	

^a^KUMC: University of Kansas Medical Center.

^b^SCr: serum creatinine.

^c^AKI: acute kidney injury.

^d^Calculated among AKI-positive patient encounters at each site.

^e^Calculated among AKI-positive patient encounters discharged alive.

^f^CVD: cardiovascular disease.

^g^CKD: chronic kidney disease.

### Model Performance on AKI Outcomes

As shown in Figure 2, BAT-AKI achieved superior discrimination on most tasks. For AKI onset prediction, BAT-AKI outperformed the second-best model by 2.8% in AUROC, 8.1% in AUPRC, and 0.3% in *F*_1_-score. For 90-day post-AKI mortality, it yielded improvements of 2.4% in AUROC, 4.1% in AUPRC, and 2.7% in *F*_1_-score over the second-best model. For AKI early reversal and recovery, BAT-AKI improved AUROC by 6% and 4.7%, and AUPRC by 4.1% and 3.7%, respectively. However, at the fixed probability threshold of .5, its *F*_1_-scores were 1.4% and 2.1% lower than those of the best-performing model for early reversal (*P*=.04) and recovery (*P*=.003), respectively. Detailed numerical results and statistical comparisons of each baseline against BAT-AKI are provided in Table S3 in Multimedia Appendix 1.

Furthermore, we evaluated the performance of BAT-AKI and baseline models on the primary task of AKI onset prediction in a low-resource setting, that is, under different fractions of the full fine-tuning dataset (1%, 2%, 5%, 15%, 25%, 50%, 75%, and 100%) to examine how performance scales with increasing training data. As shown in Figure 3, BAT-AKI achieved favorable AUROC and AUPRC beginning at 2% of the full dataset, consistently outperforming all baselines, while the transformer and XGBoost models required approximately 25% of the data to reach comparable performance. HEART, included as a pretrained EHR model benchmark, outperformed the conventional baselines in the low-resource regime but stayed below BAT-AKI at all sample sizes. With more fine-tuning data, HEART, Transformer, and XGBoost improved substantially and converged, yet still trailed BAT-AKI in both AUROC and AUPRC.

**Figure 2:**
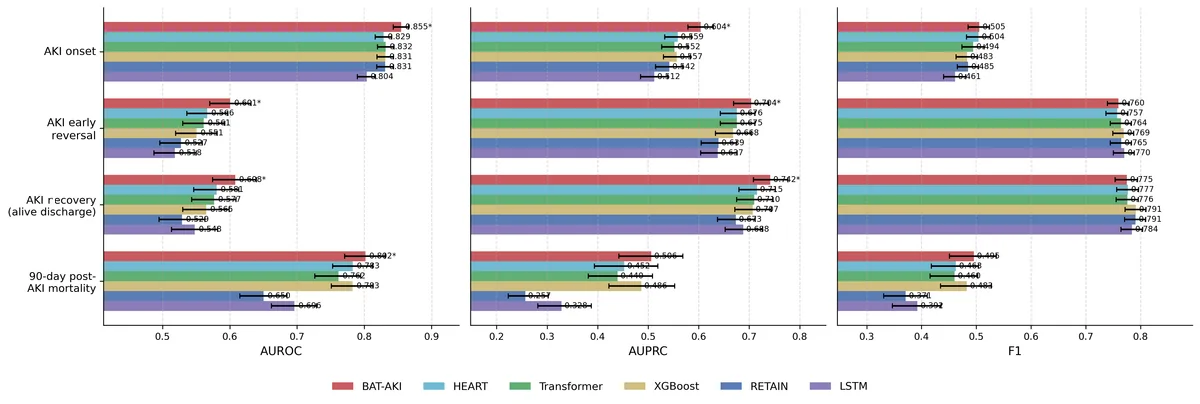
Performance of Biomarker-Aware Transformer for AKI (BAT-AKI) and baseline models on downstream tasks. Error bars denote 95% bootstrap CIs on the fixed internal test set after full fine-tuning. Asterisks indicate significant paired bootstrap comparisons between the best-performing and the second-best model for each task and metric (transformer for acute kidney injury [AKI] onset area under the receiver operating characteristic curve [AUROC] and heterogeneous relation-aware transformer [HEART] for all other starred comparisons; *P*<.05); they do not indicate significance against all baseline models. AUPRC: area under the precision-recall curve; F1: F1-score; LSTM: long short-term memory; RETAIN: Reverse Time Attention; XGBoost: extreme gradient boosting.

**Figure 3:**
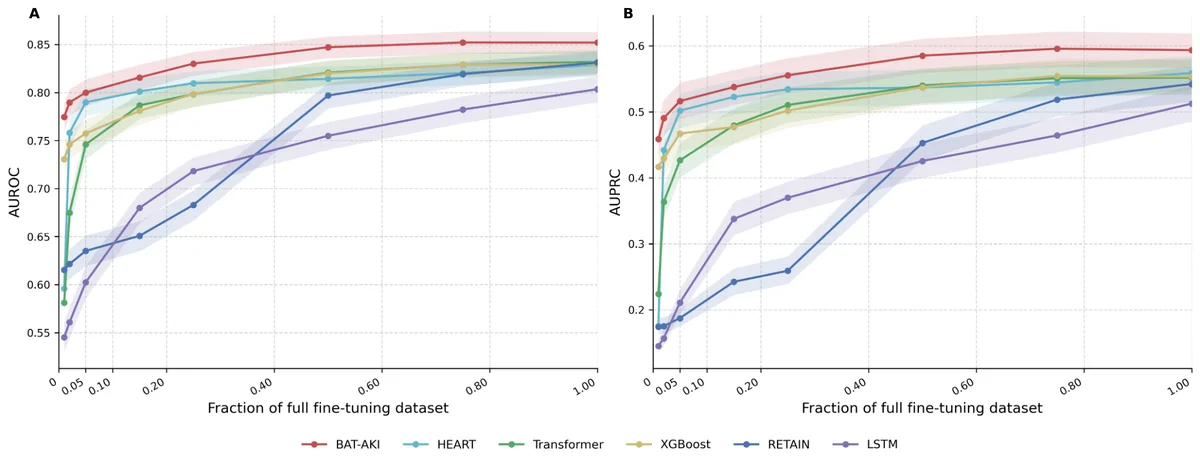
Performance scaling with increasing fine-tuning sample size for the acute kidney injury (AKI) onset prediction task. Results are evaluated on the fixed internal test set after fine-tuning with increasing fractions of the full fine-tuning pool. Error bars denote 95% CIs. Biomarker-Aware Transformer for AKI (BAT-AKI) significantly outperformed the second-best model at each sample size for both (A) area under the receiver operating characteristic curve (AUROC) and (B) area under the precision-recall curve (AUPRC) based on paired bootstrap testing. HEART: heterogeneous relation-aware transformer; LSTM: long short-term memory; RETAIN: Reverse Time Attention; XGBoost: extreme gradient boosting.

In low-resource settings (<5%), BAT-AKI achieved relative improvements of 4.2% to 6.1% in AUROC and 10.1% to 10.9% in AUPRC over the second-best model. In the moderate-scale setting (5%-25%), the relative improvements were 1.3% to 2.5% in AUROC and 2.8% to 4% in AUPRC. In high-resource settings (≥25%), BAT-AKI maintained relative improvements of 2.4% to 3.2% in AUROC and 6.2% to 8.4% in AUPRC, consistently ranking as the best-performing model across all dataset sizes.

Additional ablation studies were performed to evaluate the contribution of each embedding component and the BAP pretraining task (Method S7 in Multimedia Appendix 1). During pretraining, semantic, temporal, and ontological embeddings consistently improved overall token prediction accuracy, whereas the BAP module yielded substantially larger gains in modeling AKI-related token abnormalities and fluctuation patterns. For the primary AKI onset prediction task, all components, including the BAP module, contributed meaningfully, as removing any component resulted in significant performance degradation (Tables S4 and S5 in Multimedia Appendix 1). Sensitivity analyses comparing 3 BAP strategies—symmetric inversion, random substitution, and cyclic shift—revealed distinct effects on token-level fluctuation accuracy and downstream AKI onset performance (Method S8 and Figure S1 in Multimedia Appendix 1). Based on these results, symmetric inversion was selected as the final strategy, with additional analyses performed under varying MLM and BAP ratios (Figure S2 in Multimedia Appendix 1). We also evaluated AKI onset performance across demographic subgroups, stratified by race and age (Tables S6 and S7 in Multimedia Appendix 1). Performance differed across subgroups, with wider CIs in the smaller subgroups. The AUROC advantage was significant in the White (*P*<.001) and other (*P*=.04) subgroups but not in the Black subgroup (0.8084 vs 0.8048; *P*=.73) and was significant across all age strata. AKI incidence was 16.1% in the Black subgroup and 12.2% in the White subgroup. We further evaluated the preadmission acute kidney failure diagnosis code through sensitivity analyses (Tables S11 and S12 in Multimedia Appendix 1) and compared class weighting with equal weighting for AKI early reversal and recovery (Table S13 in Multimedia Appendix 1).

### Model Generalizability Across Multiple Clinical Datasets

As shown in Figure 4, we further compared the generalizability of BAT-AKI and the transformer baseline across different clinical datasets. For the AKI onset prediction task, BAT-AKI achieved consistent improvements over the baseline model, with AUROC gains of 2.8 to 4.0 percentage points, AUPRC gains of 6.9 to 10.7 percentage points, and *F*_1_-score gains of 1.6 to 6.6 percentage points across the 3 external validation sites. For the AKI early reversal and AKI recovery prediction tasks, the model achieved AUROC improvements of 3.5 to 6.7 percentage points and 4.5 to 5.5 percentage points, respectively, and AUPRC improvements of 2.8 to 4.9 percentage points and 3.1 to 4.1 percentage points. In the 90-day post-AKI mortality prediction task, BAT-AKI achieved AUROC improvements of 5.7 to 7.4 percentage points, AUPRC improvements of 7.7 to 12.7 percentage points, and *F*_1_-score gains of 2.0 to 6.2 percentage points across all external sites. The corresponding numerical results are summarized in Table S8 in Multimedia Appendix 1.

**Figure 4:**
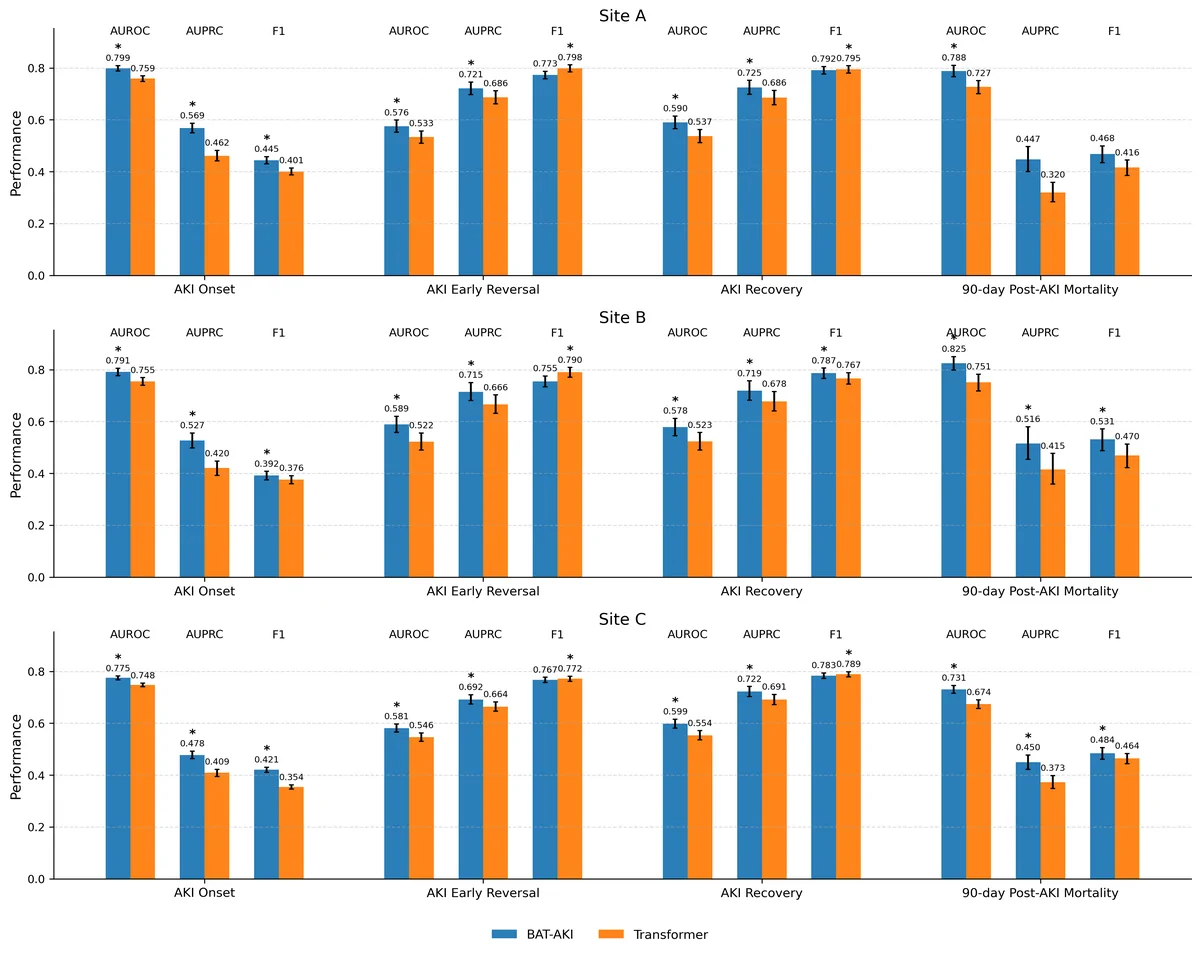
Generalizability comparison of Biomarker-Aware Transformer for AKI (BAT-AKI) and the transformer on downstream acute kidney injury (AKI) prediction tasks across multiple clinical datasets. Error bars denote 95% bootstrap CIs. Asterisks indicate significant paired bootstrap comparisons between BAT-AKI and the transformer for each metric, placed above the model with the higher value (*P*<.05). AUPRC: area under the precision-recall curve; AUROC: area under the receiver operating characteristic curve; F1: F1-score; LSTM: long short-term memory; RETAIN: Reverse Time Attention; XGBoost: extreme gradient boosting.

### Visualization of Pretraining Representations

To characterize the representation structure learned during pretraining, we visualized token-level embeddings and patient-level representations from BAT-AKI in Figure 5.

Figure 5A shows that tokens from broad clinical categories formed distinct clusters in the latent space; for example, diagnosis events were primarily distributed in the lower-central region, with finer-grained subcategories such as circulatory system diseases (*ICD-9* 390-459) forming compact clusters. Figures 5B and C display patient-level representations derived from demographic tokens, revealing 6 major patient subgroups with distinct AKI incidence rates. Cluster 2 represents a small high-risk group comprising 1.1% of the cohort with 93.3% AKI incidence, consisting of older patients with a mean age of 67.0 (SD 14.4) years whose SCr rose from a mean baseline of 0.929 (SD 0.15) mg/dL to a mean of 1.90 (SD 0.61) mg/dL at AKI onset, with 48.3% reaching stage 2 or above; clusters 4, 5, and 6 correspond to low-risk groups with AKI incidence rates of 5.7%, 7.7%, and 7.2%, respectively.

**Figure 5:**
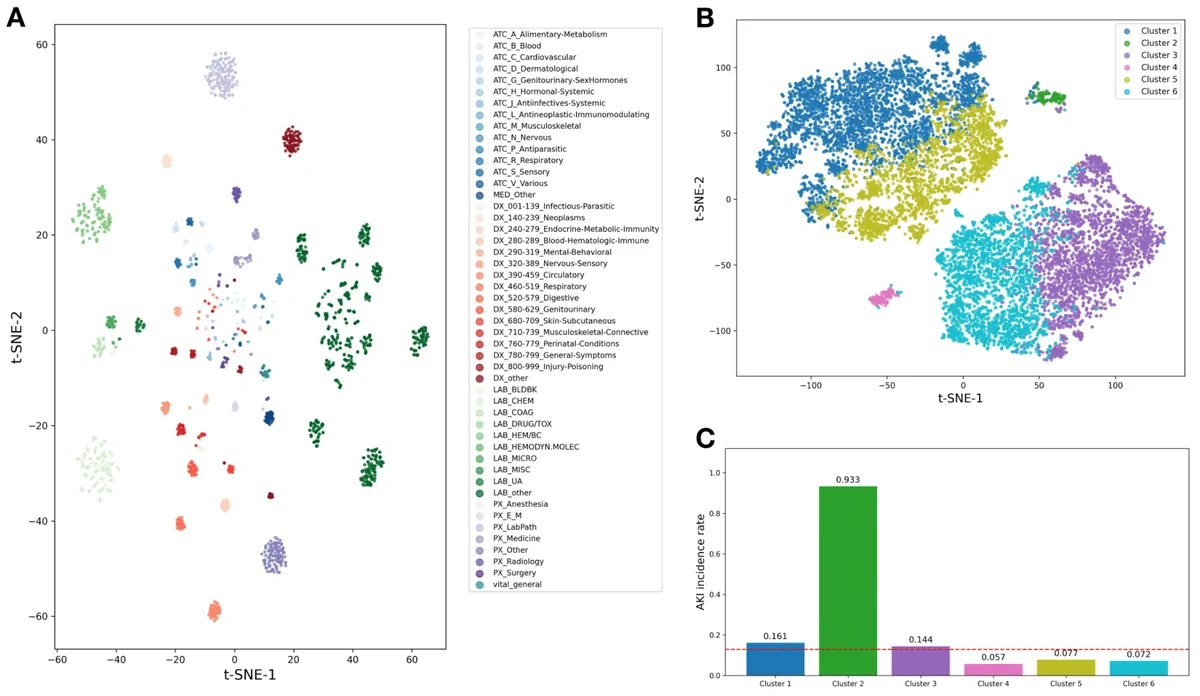
Visualization of learned embeddings and patient representations from the pretrained Biomarker-Aware Transformer for AKI (BAT-AKI) model. (A) Static token embeddings from the pretrained BAT-AKI model visualized by t-distributed stochastic neighbor embedding (t-SNE), colored according to hierarchical ontology groups. (B) Patient subgroups clustered from demographic-token hidden states of the pretrained BAT-AKI encoder visualized by t-SNE. (C) AKI incidence rates within each patient cluster, with the red dashed line indicating the average incidence rate across all patient subgroups.

### Case Study of Attention Mechanisms

We further visualized the model’s attention on a representative case (subject ID 2648, male, age 62 years) with a cardio-metabolic-renal profile, including essential hypertension, hypertensive CKD, and obesity, as shown in Figure 6. His clinical trajectory comprised 475 events over a 13-day hospitalization.

Figure 6A illustrates which earlier events the model relied on most when tracking each biomarker, showing temporal trends in potassium, blood urea nitrogen, and bicarbonate together with the top 10 most-related events for each. Potassium exhibited intermittent increases between June 30 and July 10; blood urea nitrogen showed an overall downward trend; and bicarbonate rose steadily from July 5 to July 10. For bicarbonate, on June 30, attention was driven primarily by events related to bicarbonate, anion gap, and chloride. By July 2, the pattern broadened to include sodium- and chloride-related events. By July 10, additional contributions emerged from sodium, chloride, and events linked to electrolyte and fluid disorders.

Figure 6B shows the sequential attention flow across demographic, vital sign, diagnostic, medication, and laboratory events for the same patient during the first week of hospitalization, tracing how the model connects earlier clinical events to the abnormal biomarker events that precede AKI onset. Demographic and vital events such as age, race, height, and weight directed attention toward obesity and hypertensive renal disease. As one such pathway, the medication event “Beta Blocker Selective” attended to cardio-metabolic disorder events. Laboratory measurements such as bicarbonate, chloride, and anion gap exhibited attention connections within and across time steps. Beyond these event-level attention signals, we further quantified the importance of each concept to the AKI onset prediction for this case using integrated gradients (Figure S4 in Multimedia Appendix 1). Among the highest-ranked concepts were renal-function and hemodynamic measures (SCr, blood urea nitrogen, estimated glomerular filtration rate, and diastolic blood pressure), alongside procedure and medication concepts that also ranked highly.

**Figure 6:**
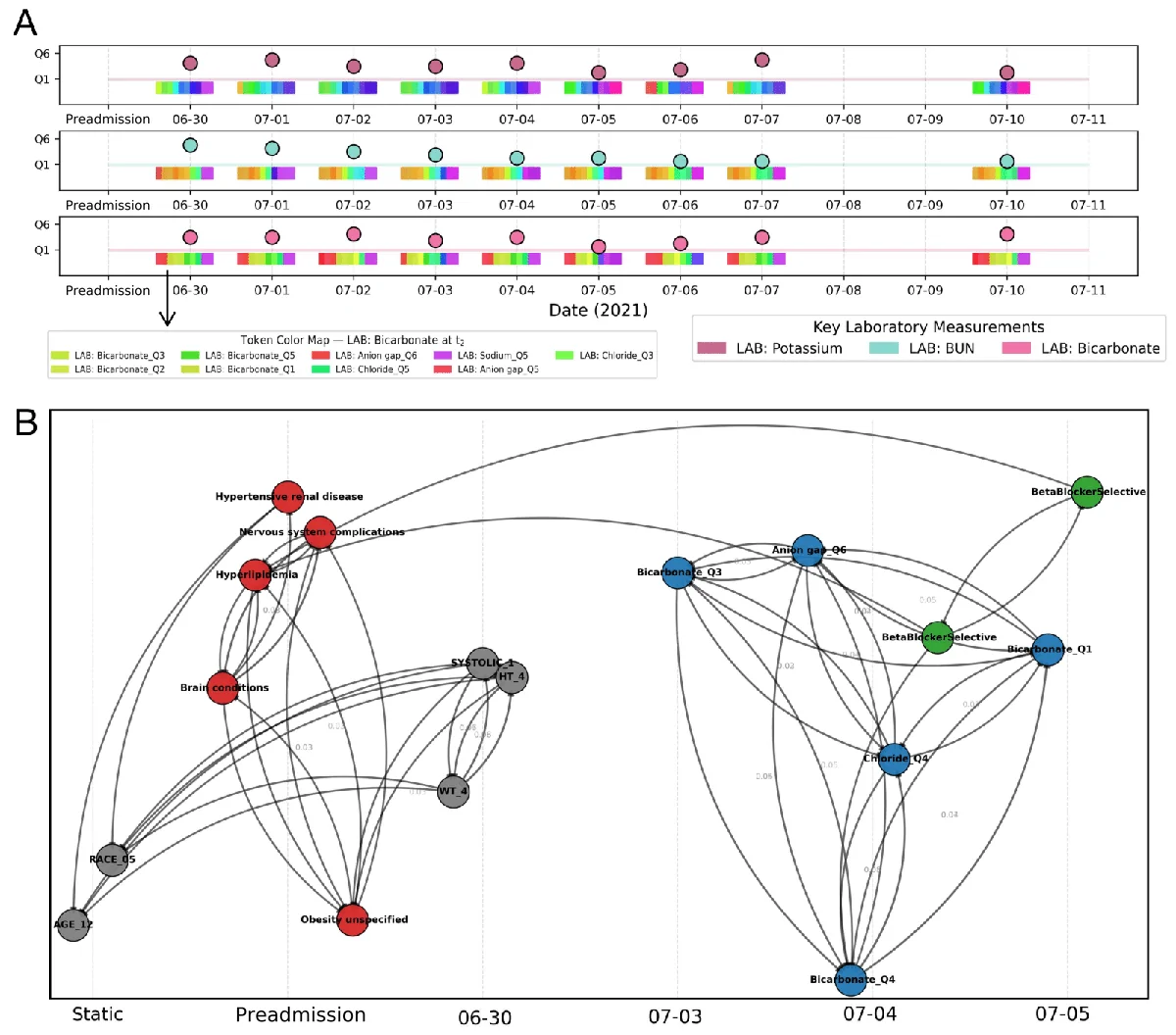
Visualization of attention patterns from the pretrained Biomarker-Aware Transformer for AKI (BAT-AKI) model on a representative case (subject ID 2648). (A) Temporal trends of 3 laboratory measurements and the top-10 events most related to each. For each measurement, these are the earlier events the model linked to it most strongly. The detailed color assignments for all events are provided in Figure S3 in Multimedia Appendix 1. (B) Visualization of the most-attended events identified by the model across multiple time steps during the first week of hospitalization. Each point is a clinical event, and events are arranged left-to-right along the hospital timeline, so the chain can be followed as the hospitalization unfolds. Each arrow connects a pair of events that the model linked through attention, running from the attending event to the event it attends to. Colors indicate the type of event. Only arrows with attention scores greater than 0.001 are displayed, and those with scores greater than 0.02 are annotated with their score. BUN: blood urea nitrogen.

## Discussion

### Main Findings

In this study, we developed BAT-AKI, a transformer-based framework that couples multisource embeddings with a BAP objective, and showed that a single pretrained model can be fine-tuned across the AKI course—from onset prediction to postonset prognosis—while providing token- and patient-level interpretability insights.

Our results highlight the importance of both multisource embedding design and the BAP pretraining objective. As shown in Figure 1B, incorporating ontology information improved the hierarchical structure of token representations (Figure 5A), strengthening token-level (ie, individual clinical event) attention patterns, while textual embeddings enriched semantic context and improved pretraining performance and downstream AKI onset prediction (Table S4 in Multimedia Appendix 1). The BAP objective increased model sensitivity to AKI-related fluctuations and improved abnormal-token detection accuracy from 0.7595 to 0.9178, a 21% relative improvement, leading to consistently better performance across all 4 downstream tasks compared with models pretrained without BAP (Table S5 in Multimedia Appendix 1). These findings underscore the value of incorporating task-relevant signals into pretraining for EHR modeling.

Across downstream prediction tasks, BAT-AKI demonstrated strong performance and adaptability, consistently outperforming baseline models in AUROC and AUPRC with simple fine-tuning (Figure 2; Table S3 in Multimedia Appendix 1), with the largest relative gains on the lower-prevalence postonset prognostic tasks. However, improvements in AUROC and AUPRC did not uniformly translate into higher *F*_1_-scores at the fixed probability threshold of .5, underscoring that *F*_1_-score performance depends on the selected classification threshold. In low-resource settings, BAT-AKI reached, with only 2% of the fine-tuning data, a level of performance that HEART required 5% and the transformer and XGBoost approximately 25% of the data to match (Figure 3). These findings suggest that pretraining provides a robust initialization that enables efficient adaptation to diverse AKI-related prediction tasks, particularly when labeled data are limited.

BAT-AKI also demonstrated superior cross-site generalizability compared with the transformer baseline. As shown in Figure 4 and summarized in Table S8 in Multimedia Appendix 1, across the external validation sites, BAT-AKI consistently achieved higher AUROC and AUPRC, indicating improved robustness to site-level heterogeneity. This suggests that large-scale pretraining enables learning of transferable and domain-invariant representations, whereas models trained solely on site-specific fine-tuning data may be more susceptible to performance degradation in external settings.

Finally, to understand how BAT-AKI encodes clinical knowledge, we examined its embedding space and attention patterns through pretraining visualizations and a case study. Token embeddings formed coherent clusters reflecting the hierarchical ontology structure (Figure 5A), and ablation confirmed that removing any embedding component reduced token-prediction accuracy and degraded AKI onset performance (Method S7 and Table S4 in Multimedia Appendix 1), indicating that the embedding layer captures semantic and structural relationships beyond token identity. Demographic-based representations alone separated patients into subgroups with distinct AKI incidence (Figures 5B and C), while attention shifted across related laboratory tokens to track temporal biomarker patterns (Figure 6A) and integrated information across demographics, laboratory tests, diagnoses, and medications within a patient’s trajectory (Figure 6B). Consistent with these attention patterns, integrated-gradient attributions highlighted clinically relevant renal, physiologic, and treatment-context concepts—including SCr, blood urea nitrogen, and blood-pressure measurements at the case level, with medication-exposure concepts more prominent at the cohort level (Figure S5 in Multimedia Appendix 1). Together with the attention analysis, these results indicate that, after task-specific fine-tuning, the pretrained model bases its AKI onset predictions on clinically interpretable features.

### Limitations

Despite these promising results, some limitations warrant consideration. First, the AKI onset definition relies entirely on SCr changes, and the official urine output criteria were omitted because urine output was not consistently captured in the EHR across sites; as a result, a subset of AKI cases may not have been captured. Second, mortality data were obtained by linking patient records to the Social Security Administration’s Death Master File; while this source captures all reported deaths, delayed or incomplete reporting and limited follow-up remain possible, potentially leading to some deceased patients being misclassified as survivors. Third, some laboratory input tokens, including race-adjusted estimated glomerular filtration rate, reflect measurements as they were historically recorded in the retrospective EHR data and may carry biases from prior clinical practice. Fourth, the statistical selection of the BAP lexicon was performed without correction for multiple comparisons. The impact of this choice was limited because retained concepts were subsequently filtered against prior clinical evidence and used only to define perturbation targets during pretraining rather than model input features. Fifth, the XGBoost baseline encodes the presence of clinical events across the observation window but not their temporal ordering, and richer temporal feature engineering could narrow the gap. Sixth, HEART was fine-tuned using its native pretrained-model configuration rather than the validation-based tuning used for BAT-AKI and the conventional baselines, which may have disadvantaged HEART in the comparison. Seventh, the cross-site analysis was limited to BAT-AKI and the structurally equivalent transformer baseline and did not include HEART, limiting the generalizability conclusions to this comparison. Eighth, the discrimination for the early reversal and recovery tasks remains modest, reflecting the inherent difficulty of predicting these post-AKI trajectories from preonset information alone, which could be improved by incorporating postonset information as the hospitalization evolves. Ninth, our interpretability analyses rely on embedding visualization, attention weights, and integrated-gradient attributions, which provide useful but indirect evidence of model behavior. Tenth, this study is based on retrospective EHR data and does not yet evaluate prospective clinical deployment or real-time decision support. As an initial assessment of clinical applicability, we report calibration and decision-curve analyses for the AKI onset task in Figures S6 and S7 in Multimedia Appendix 1, where net benefit was positive only after Platt recalibration; realizing this in practice will require further prospective validation and clinical implementation. After recalibration, differences in net benefit between BAT-AKI and the benchmark models were on the order of 0.005 to 0.01. Platt scaling was fitted and evaluated within the internal cohort only, and site-specific recalibration would be required prior to any deployment. Eleventh, BAT-AKI’s advantage over the transformer was reduced in the Black subgroup, where the AUROC difference was not significant. Absolute AUROC values are not directly comparable across subgroups given the differences in AKI incidence and subgroup size, and subgroup-specific evaluation would be needed before clinical use. Ensuring fairness across demographic and socioeconomic groups is an important direction for future work, which may benefit from more extensive adaptation methods to address the performance differences observed across subgroups. Future work will focus on prospective validation, updated outcome definitions incorporating urine output, fairness analyses across patient subgroups, and richer temporal and postonset modeling of post-AKI trajectories to more comprehensively assess and improve the model’s real-world utility.

### Conclusions

In conclusion, BAT-AKI is a pretraining-fine-tuning framework for AKI prediction and prognosis that demonstrates competitive predictive performance, cross-institution generalizability, and interpretability insights into its predictions. By leveraging transformer-based representations tailored to longitudinal EHR data, a single pretrained model supports multiple AKI-related tasks spanning early prediction and postonset prognosis. These findings suggest that BAT-AKI provides a scalable and generalizable foundation for EHR-based AKI risk modeling, with potential to support early warning, patient stratification, and downstream clinical decision-making in real-world health care settings.

## Data Availability

The data used in this study were deidentified EHRs from the GPC and PaTH networks within the National Patient-Centered Clinical Research Network (PCORnet), supported by PCORI. Due to data-use agreement (DUA) restrictions governing the use and sharing of these data, the authors are unable to share individual-level data outside of the study team. Qualified researchers may request access to GPC data by submitting a formal application through the GPC Query and Data Request Form. All data requests are reviewed by the GPC Data Request Oversight Committee (DROC), the Research Opportunity Assessment (ROA) Committee, and local institutional representatives to ensure compliance with governance, IRB, and data-use policies. Similarly, access to PaTH data is available to qualified researchers through the PaTH data request and governance process, subject to approval by the PaTH network and participating institutions in accordance with applicable IRB and data-use requirements. Detailed information about the GPC and PaTH networks is available on their respective websites [43,44]. Additional information about PCORnet is available on its website [45]. Detailed implementation and source code for this study are available at GitHub [46].

## Appendix

Multimedia Appendix 1 Additional methods, tables, and figures for the development, evaluation, sensitivity analyses, and interpretability analyses of the Biomarker-Aware Transformer for Acute Kidney Injury (BAT-AKI).

## Abbreviations

AKI: acute kidney injury
ATC: Anatomical Therapeutic Chemical Classification System
AUPRC: area under the precision-recall curve
AUROC: area under the receiver operating characteristic curve
BAP: biomarker abnormality-aware pretraining
BAT-AKI: Biomarker-Aware Transformer for AKI
CKD: chronic kidney disease
CPT: Current Procedural Terminology
EHR: electronic health record
FFN: feed-forward network
GPC: Greater Plains Collaborative
HEART: heterogeneous relation-aware transformer
HIPAA: Health Insurance Portability and Accountability Act
ICD: International Classification of Diseases
KUMC: University of Kansas Medical Center
LN: layer normalization
LOINC: Logical Observation Identifiers Names and Codes
MHSA: multihead self-attention
MLM: masked language modeling
PCORnet: the National Patient-Centered Clinical Research Network
RETAIN: Reverse Time Attention
SCr: serum creatinine
t-SNE: t-distributed stochastic neighbor embedding
UMLS: Unified Medical Language System
XGBoost: extreme gradient boosting
