# Supplementary material for: Early Prediction and Prognosis in Acute Kidney Injury Using a Biomarker-Aware Transformer: Retrospective Multicenter Study
Source: JMIR Med Inform. 2026 Oct 26;14:e90687. doi: 10.2196/90687 (PMC13749021; doi:10.2196/90687)
Supplement: Supplementary file 1 [file medinform_v14i1e90687_app1.docx]

**Table of Contents**

**1. Method S1. Definition of Downstream Tasks**
1.1 AKI onset
1.2 AKI reversal (RVRT / ERVRT)
1.3 AKI recovery (RCV)
1.4 90-day post-AKI mortality (Death90)

**2. Method S2. EHR Data Preprocessing, Standardization, and Quantile-Based Tokenization**
2.1 Data standardization
2.2 Quality control and filtering
2.3 Tokenization and temporal ordering of clinical events

**3. Method S3. Model Embedding**
3.1 Semantic embedding generation
3.2 Ontology embedding

**4. Method S4. Construction of the AKI-Related Lexicon (V_aki)**

**5. Method S5. Implementation Details of BAT-AKI**
5.1 Pretraining configuration
5.2 Fine-tuning and baseline models

**6. Method S6.** **Interpretability Analysis Procedures**
6.1 Token embedding analysis
6.2 Case study
6.3 Attention-flow graph construction

**7. Method S7.** **Ablation Study**
7.1 Embedding component ablations
7.2 Effect of abnormal token injection (BAP)

**8. Method S8.** **Model Sensitivity Analysis**

**9. Tables**
**Table S1.** Method pre-configuration parameters
**Table S2.** ICD codes for sub-population definitions
**Table S3.** Downstream task performance
**Table S4.** Embedding component ablation
**Table S5.** BAP ablation
**Table S6.** AKI-onset performance across race subgroups
**Table S7.** AKI-onset performance across age subgroups
**Table S8.** Cross-site generalizability
**Table S9.** MDRD baseline sensitivity analysis
**Table S10.** Retained clinical concepts in the AKI-related lexicon

**Table S11.** Frequency of the acute kidney failure diagnosis code among preadmission records
**Table S12.** Sensitivity analysis after removing the preadmission acute kidney failure diagnosis code from the AKI-onset input sequences
**Table S13.** Comparison of weighting strategies for BAT-AKI on AKI early reversal and recovery

**10. Figures**
**Figure S1.** Comparison of BAP strategies
**Figure S2.** Symmetric BAP strategy under different masking/perturbation ratios
**Figure S3.** Complete color mapping for fluctuation-trajectory visualizations

**Figure S4.** Concept importance, case study (integrated gradients)

**Figure S5.** Concept importance, cohort level (integrated gradients)

**Figure S6.** Calibration curves

**Figure S7.** Decision curves

**11. References**

**Method S1. Definition of downstream tasks**

**1. AKI Onset**
Acute kidney injury (AKI) onset was identified according to KDIGO criteria^1^: either an increase in serum creatinine (SCr) by ≥0.3 mg/dL within 48 hours, or an increase to ≥1.5 times the baseline within 7 days. Baseline SCr was estimated hierarchically using the most recent SCr within 7 days prior to admission, or the mean SCr from 7–365 days prior to admission when recent values were unavailable, with MDRD back-calculation applied if no prior measurements existed^2^. AKI onset was defined using the serum creatinine criteria; the urine output criterion was not incorporated. We conducted a sensitivity analysis for the MDRD back-calculation by excluding encounters whose baseline SCr required this imputation. In the fine-tuning and test cohorts, AKI incidence before versus after exclusion was 12.64% versus 12.98% at KUMC, 15.57% versus 16.05% at Site A, 15.60% versus 15.64% at Site B, and 14.18% versus 14.46% at Site C. Because the exclusion also reduced the size of the downstream data, BAT-AKI was fine-tuned and evaluated on the MDRD-excluded cohort and compared with a control cohort from which an equal number of randomly selected encounters were removed, holding the pretrained encoder fixed; AKI-onset performance did not differ significantly between the two runs (**Table S9**).

**2. AKI Reversal**
AKI reversal was defined as the occurrence of a KDIGO-defined reversal during hospitalization, i.e., SCr declining below AKI thresholds and remaining for ≥1 day after onset. Early reversal (ERVRT) was further defined as reversal within 7 days of AKI onset.

**3. AKI Recovery**
AKI recovery was defined at the encounter level by the last available SCr before discharge. Recovery was assigned if the final SCr value satisfied KDIGO reversal thresholds (<1.5× baseline or <0.3 mg/dL above reference), indicating sustained improvement at the end of hospitalization. Patients who died during the index hospitalization were therefore not included in this task, avoiding conflation of in-hospital mortality with persistent kidney dysfunction.

**4. 90-day post-AKI mortality**
90-day post-AKI mortality was defined as all-cause death within 90 days following AKI onset.

**Method S2. EHR data preprocessing, standardization, and quantile-based tokenization**

**1. Data standardization**

Diagnosis, procedure, laboratory, and medication codes were standardized across sites to ensure consistency of medical concepts.

**Diagnoses** were unified to the International Classification of Diseases, Ninth Revision (ICD-9) system. Codes originally in ICD-10 were mapped to ICD-9 using the official General Equivalence Mappings. When no direct equivalence existed, the original ICD-10 code was retained to preserve information integrity. These retained ICD-10 tokens were assigned to a general category for other diagnoses, and were rare (1.16% of diagnosis token types). **Procedures** were represented using Current Procedural Terminology (CPT) codes. **Laboratory measurements** were standardized across local systems using Logical Observation Identifiers Names and Codes (LOINC). Units were harmonized to the canonical LOINC units. When applicable, tests were additionally mapped to their corresponding LOINC Group (LG) identifiers to facilitate unique variable representation during modeling. Laboratory items without valid LG or unit conversions retained their original identifiers. **Medications** were integrated using both Anatomical Therapeutic Chemical (ATC) and RxNorm terminologies. RxNorm concepts were converted to their corresponding ATC classes whenever possible; medications without a valid ATC mapping were kept under their original RxNorm identifiers.

**2. Quality control and filtering criteria**

Two levels of quality control were applied to ensure data reliability and model robustness.

**Diagnosis granularity.** The ICD-9 diagnosis codes were evaluated at both 3-digit and 5-digit levels. Considering the balance between data coverage and code specificity, we adopted the 3-digit granularity as the final representation. In addition, recurrent diagnoses preceding the index admission were differentiated by temporal proximity, with events occurring within or beyond six months represented separately to incorporate short-term versus long-term disease information.

**Variable prevalence threshold.** For all clinical variables (diagnoses, laboratory tests, medications, procedures, and vitals), we examined different inclusion thresholds based on their population prevalence (0.05%, 1%, 5%, and 10%). Finally, a 1% cutoff was applied, meaning that variables appearing in fewer than 1% of encounters were excluded. This threshold provided an optimal balance between retaining informative features and mitigating data sparsity, resulting in stable model performance.

**3. Tokenization and temporal ordering of clinical events**

To construct tokenized event sequences for model input, all clinical observations were converted into discrete tokens representing distinct medical events. Continuous measurements, including laboratory tests and vital signs, were quantized to enable uniform categorical representation across variables. Based on empirical value distributions computed from the pretraining set, laboratory measurements and vital signs were discretized into six quantile bins (Q1–Q6). Each quantile bin was encoded as a categorical token reflecting the relative level of the measurement. Demographic variables were encoded as categorical tokens: age was discretized into 20 equal-width bins spanning the adult age range observed in the pretraining cohort, each encoded as an ordered age token, and each sex and race category was encoded as its own categorical token.

The resulting clinical events—including demographic characteristics, vital signs, diagnoses, procedures, laboratory measurements, and medications—were organized into chronological sequences. Events were ordered by their recorded timestamps and, when multiple events occurred at the same time, by their clinical category in a predefined order to preserve consistent temporal and contextual structure for modeling.

When the resulting event sequence exceeded the maximum length M = 500, demographic and diagnosis tokens were always retained, preserving each patient's baseline characteristics and diagnosis history. The sequence was then filled up to M = 500 with the most recent events, dropping the earliest, since events closest to the end of the observation window are expected to be most relevant to AKI onset and post-AKI risk.

**Method S3. Model embedding**

**1. Semantic embedding generation**

For semantic embedding, we used code descriptions from standard medical terminology systems, including ICD, CPT, LOINC, RxNorm, and ATC. We compared the vector representations generated by two large language models: **Sentence-BERT and BioBERT. After comprehensive evaluation, we** selected Sentence-BERT because it achieved better performance in the pretraining task**.** To maintain consistency with our model architecture, we further reduced its dimensionality and set the semantic-embedding dimension to 128.

**2. Ontology embedding**

We primarily utilized two types of information in ontology embedding: hierarchical information (hontology) and semantic type information (sontology). First, hierarchical information was based on the hierarchical structure of each code system (ICD, CPT, LOINC, RxNorm, and ATC). For consistency, we used the highest-level category of each code in its system as its hierarchical representation. For example, code ICD-250.00 belongs to 240–279 Endocrine, Nutritional and Metabolic Diseases, and Immunity Disorders in the ICD system, so this superclass serves as its hontology representation. Second, semantic type information was derived from the UMLS system, which provides unified semantic type labels for different types of medical codes, used to describe the semantic interactions between different codes. We directly used the semantic types provided by UMLS as categories in sontology. If a code belongs to multiple semantic types under the UMLS system, we treated each combination “A and B” as a separate category.

**Method S4. Construction of the AKI-related Lexicon (**$\boldsymbol{V}_{\boldsymbol{aki}}$**)**

Candidate concepts spanned laboratory measurements, medications, procedures, diagnoses, and vital signs. On a dedicated exploration split of the KUMC pretraining data, held out for this purpose prior to pretraining, we used encounter-level token-frequency differences to identify AKI-associated EHR events that could guide abnormality-aware perturbation. Quantile variants of continuous laboratory and vital-sign tokens were collapsed to the concept level before testing. For each candidate concept *c*, we computed its per-encounter relative frequency *f_i,c_* = *n_i,c_* / *L_i_*, where *n_i,c_* is the number of occurrences of concept *c* in encounter *i* and *L_i_* is the total sequence length of that encounter. We compared *f_i,c_* between AKI-positive and AKI-negative encounters using a two-sided Mann-Whitney U test, retaining concepts with *P*<.05. The relative frequency *f*_i,c_ measures each concept's share of the encounter's clinical record, and both enriched and depleted concepts were retained. Prior evidence on AKI risk factors and predictors^3-5^ was then applied to remove concepts without an established connection to the inpatient AKI care pathway.

The final *V_aki_* contained 119 clinical concepts, including 51 laboratory, 24 medication, 30 procedure, 12 diagnosis, and 2 vital-sign concepts. The retained concepts are listed in **Table S10**. The frequency of the preadmission acute kidney failure diagnosis DX:09:584 and the sensitivity analysis after removing this code from the AKI-onset input sequences are reported in **Tables S11 and S12**, respectively.

**Method S5. Implementation details of BAT-AKI**

Hyperparameter selection was performed only within the KUMC development cohort using the held-out validation split; the fixed temporal test set and external validation cohorts were not used for model selection. For BAT-AKI pretraining, candidate configurations were evaluated over the number of Transformer layers, attention heads, embedding dimension, dropout rate, and learning rate. The final configuration was selected based on validation performance and computational efficiency, then fixed before downstream evaluation. For downstream fine-tuning, the prediction-head structure and optimization settings were selected on the AKI-onset validation task and then applied uniformly across downstream tasks to avoid task-specific over-tuning. Neural baselines were trained under aligned optimization settings whenever applicable, including batch size, learning rate, validation-based early stopping, and maximum training epochs. HEART^6^ was fine-tuned using its native pretrained-model configuration, with a larger epoch budget and the same validation-based early-stopping strategy used for BAT-AKI. XGBoost was tuned on the same validation split. All final evaluations were performed once on the fixed internal temporal test set and external cohorts.

**1. BAT-AKI Pretraining**

Pretraining sequences were constructed using the same pre-onset observation window as the downstream model inputs. BAT-AKI was pretrained using a parameter search pool, from which the final configuration was selected by coordinate-wise selection over approximately 13-15 pretraining runs:

- Transformer layers: chosen as 2 (candidates = {1, 2, 4})
- Attention heads: chosen as 4 (candidates = {1, 2, 4})
- Embedding dimension: 256 (candidates = {128, 256, 512, 1024})
- Hidden size (feed-forward dimension): 2 × embedding dimension
- Dropout: 0.1 (candidates = {0.1, 0.3, 0.5})
- Learning rate: 1e-4 (candidates = {1e-5, 2e-5, 1e-4, 1e-3})
- Batch size: 128
- Optimization: Adam optimizer with early stopping (patience = 10 epochs)

**2. Fine-tuning and Baselines**

BAT-AKI and all baseline deep learning models shared consistent training hyperparameters to ensure fair comparison. Specifically, all downstream models were trained with a hidden dimension of 256, dropout rate of 0.1, batch size of 128, learning rate of 1×10⁻⁴, and an early-stopping patience of 10 epochs. The LSTM, vanilla Transformer, and RETAIN were implemented using the PyHealth package, with hidden dimensions aligned to BAT-AKI for fair comparison.

For XGBoost, we used a tabular representation built from the same observation-window tokens as the deep learning models, in which each feature indicates whether a clinical concept occurred at any point within the window. This representation covers the same event history available to the sequence models but does not preserve temporal ordering. Sparse features with a frequency below 0.05 were dropped to ensure more stable inputs. Hyperparameters were selected by grid search over the following candidate pool, with validation-based early stopping: n_estimators=600 (candidates = {100, 200, 300, 400, 500, 600, 700, 800}), max_depth=3 (candidates = {3, 4}), γ=1 (candidates = {0, 1}), subsample=0.8 (candidates = {0.6, 0.8, 1}), colsample_bytree=0.8 (candidates = {0.6, 0.8, 1}).

For the imbalanced AKI-onset and 90-day post-AKI mortality tasks, class weighting was applied using $w_{\mathrm{pos}}={N_{\mathrm{neg}}}/{N_{\mathrm{pos}}}$, with the negative class weight fixed to 1. Equal class weighting ($w_{\mathrm{pos}}=1$) was used for AKI early reversal and recovery based on validation performance. Model training and early stopping were based on the held-out validation split, and final performance was evaluated on the fixed internal temporal test set. Results obtained using $w_{\mathrm{pos}}={N_{\mathrm{neg}}}/{N_{\mathrm{pos}}}$ for early reversal and recovery are reported in **Table S13**. F1-scores were calculated using a fixed probability threshold of 0.5.

All experiments were implemented in PyTorch (v2.1.2) with CUDA 12.1. BAT-AKI was pretrained on two NVIDIA L4 GPUs and fine-tuned on a single NVIDIA L4 GPU, consistent with all baseline models.

**Method S6. Interpretability analysis procedures**

1. Token embedding analysis: Static token embeddings were concatenated across all static components after normalization and projected into a two-dimensional space using t-SNE (perplexity = 35, n_iter = 4000) for visualization. Patient-level embeddings were derived from the demographic-token representations of the pre-trained model and reduced to 50 dimensions using PCA. The vectors were then clustered into six patient subgroups using spectral clustering (n_clusters = 6, n_neighbors = 20) and projected into two dimensions via t-SNE (perplexity = 30, n_iter = 4000) for visualization. Cluster-level statistics, including the prevalence of AKI onset, were summarized to characterize subgroup differences.

2. Case study: A two-stage case study was implemented on a representative patient (ID = 2648) to visualize token-level attention patterns. In Part 1, we extracted attention weights from the final Transformer layer and examined how the model attended to several key laboratory measurements (blood urea nitrogen, bicarbonate, potassium). For each laboratory token with high quantile levels, we computed its top-10 most attended source categories by averaging multi-head attention scores across valid sequence positions. Each token and its attended sources were semantically mapped to clinical categories (laboratory tests, medications, diagnoses, and procedures) using the expanded code dictionary. The resulting top-attention relationships were then visualized along the clinical timeline to reveal how different clinical factors dynamically influenced these key laboratory measurements. In Part 2, we further analyzed token-level attention relationships to examine how the model captured interactions among heterogeneous clinical events. To effectively illustrate inter-token relationships within limited space, tokens were selected according to their clinical semantics, cross-type relevance, and temporal coverage, focusing on those with the strongest incoming and outgoing attentions across different code types. To reduce noise, both attention-in and attention-out thresholds were set at 0.01. The resulting subset was used to construct a directed attention-flow graph, revealing cross-category and cross-time connections that reflect how BAT-AKI integrates heterogeneous clinical information across different event types and temporal contexts.

**3. Integrated-gradients feature attribution:** We applied integrated gradients (IG)^7^ to estimate concept-level feature importance for AKI-onset prediction. Token variants corresponding to the same clinical concept (e.g., temporal-window, laboratory quantile, and continuous-value bin variants) were collapsed, and signed token-level attributions were aggregated within each encounter:

$$\varphi_{c}\left( x \right)=\sum_{t: g\left( t \right)=c} \mathrm{IG}_{t}\left( x \right),$$

where $g\left( t \right)$ denotes the clinical concept associated with token position $t$. Concept importance was then defined as the mean absolute encounter-level attribution among test encounters containing that concept:

$$\mathrm{Importance}\left( c \right)=\frac{1}{\left| D_{c} \right|}\sum_{x\in D_{c}} \left| \varphi_{c}\left( x \right) \right|,$$

where $D_{c}$ denotes the subset of test encounters containing concept $c$. Concepts were ranked by their mean absolute attribution, which reflects magnitude alone.

Method S7. Ablation Study

To better understand the contribution of each component in **BAT-AKI**, we performed two sets of ablation experiments as following.

**1. Embedding ablations.**
We sequentially removed each embedding channel—semantic, temporal, type, hierarchical ontology, and semantic ontology—from the pretraining configuration. For each variant, we evaluated both the **Top-1 token prediction accuracy** during pretraining and the downstream performance on AKI onset prediction (AUROC, AUPRC, F1-score).

**2. Effect of abnormal token injection.**
We further evaluated the contribution of the biomarker abnormality-aware pretraining (BAP) task by assessing two complementary capabilities:(a) token-level fluctuation robustness, and (b) abnormal position detection. For (a), we measured the model’s ability to correctly recover masked tokens that represent highly fluctuating AKI-related events—including (i) laboratory biomarkers exhibiting strong within-patient quantitative variability and (ii) categorical clinical events (e.g., medications, procedures, diagnoses) showing large frequency shifts relative to population-level patterns. For (b), we evaluated the model’s ability to identify abnormal biomarker positions, i.e., whether each token corresponds to an abnormal (flag = 1) or normal (flag = 0) state, as predicted by the model’s abnormality detection head. Together, these two evaluations characterize the model’s robustness to clinically meaningful fluctuations and its capability to recognize abnormal biomarker states.

Method S8. Model sensitivity analysis

This analysis evaluates the model’s sensitivity to different abnormal perturbation strategies and hyperparameter configurations. Two dimensions were examined. First, we compared three BAP strategies—symmetric, random, and cyclic—to assess how different abnormal token perturbation mechanisms influence the model’s ability to capture AKI-related token dynamics. Second, we varied the masked language modeling (MLM) masking ratio and BAP perturbation ratio under the best-performing symmetric strategy to examine how these hyperparameters jointly affect model stability and representational performance.

The evaluation focuses on fluctuation prediction accuracy and fluctuation detection accuracy. The fluctuation prediction accuracy measures the model’s ability to correctly reconstruct fluctuating AKI-related tokens—tokens whose quantile-level variance differs significantly between AKI and non-AKI patients in the test set, such as laboratory measurements that reflect renal function changes. The fluctuation detection accuracy reflects the capability of the model’s embedding representation and abnormal prediction head, trained during pretraining, to correctly identify whether these fluctuating tokens are in an abnormal (flag = 1) or normal (flag = 0) state.

Additionally, onset AUROC and onset AUPRC represent the area under the ROC and precision–recall curves for the downstream AKI onset prediction task, providing a complementary measure of clinical outcome prediction.

| The maximum length $M$ | 500 |
| --- | --- |
| Total number of downstream tasks $s$ | 4 |
| $\rho_{MLM}$ | 0.15 |
| $\rho_{BAP}$ | 0.3 |
| $L$ | 2 |
| $Q$ | 6 |
| λ | 1 |

**Table S1.** Method pre-configuration parameters.

|  | ICD-9 | ICD-10 |
| --- | --- | --- |
| Cancer | 140–208, 230–234 | **C00–C97**, **D00–D09** |
| Cardiovascular Disease | 390–459 | I00–I99 |
| Chronic Kidney Disease | 585 | N18 |
| Diabetes Mellitus | 250 | E08–E13 |
| Hypertension | 401–405 | I10–I15 |

**Table S2.** ICD codes used to define sub-populations.

| **(A) Task: AKI Onset** | | | | **(B) Task: AKI Early Reversal** | | | |
| --- | --- | --- | --- | --- | --- | --- | --- |
| **Model** | **AUROC** | **AUPRC** | **F1** | **Model** | **AUROC** | **AUPRC** | **F1** |
| BAT-AKI | 0.8549 (0.8433–0.8658) Reference | 0.6041 (0.5786–0.6292) Reference | 0.5054 (0.4849–0.5239) Reference | BAT-AKI | 0.6006 (0.5703–0.6318) Reference | 0.7036 (0.6690–0.7388) Reference | 0.7596 (0.7394–0.7790) Reference |
| HEART | 0.8288 (0.8164–0.8409) P<.001 | 0.5587 (0.5329–0.5856) P<.001 | 0.5041 (0.4821–0.5253) P=.88 | HEART | 0.5664 (0.5362–0.5973) P=.006 | 0.6758 (0.6426–0.7114) P=.02 | 0.7573 (0.7367–0.7768) P=.64 |
| Transformer | 0.8319 (0.8197–0.8442) P<.001 | 0.5518 (0.5267–0.5785) P<.001 | 0.4941 (0.4736–0.5143) P=.16 | Transformer | 0.5610 (0.5301–0.5927) P=.007 | 0.6754 (0.6426–0.7115) P=.06 | 0.7644 (0.7447–0.7832) P=.27 |
| RETAIN | 0.8311 (0.8187–0.8435) P<.001 | 0.5420 (0.5148–0.5685) P<.001 | 0.4848 (0.4656–0.5041) P=.002 | RETAIN | 0.5272 (0.4958–0.5593) P<.001 | 0.6392 (0.6050–0.6751) P=.002 | 0.7647 (0.7448–0.7832) P=.27 |
| LSTM | 0.8036 (0.7897–0.8167) P<.001 | 0.5123 (0.4850–0.5391) P<.001 | 0.4610 (0.4405–0.4810) P<.001 | LSTM | 0.5183 (0.4873–0.5503) P<.001 | 0.6375 (0.6041–0.6759) P=.001 | 0.7702 (0.7504–0.7884) P=.04 |
| XGBoost | 0.8313 (0.8189-0.8437) P<.001 | 0.5566 (0.5301-0.5838) P<.001 | 0.4829 (0.4632-0.5022) *P*=.004 | XGBoost | 0.5506 (0.5195–0.5813) P=.003 | 0.6681 (0.6333–0.7043) P=.03 | 0.7692 (0.7494–0.7875) P=.08 |
| **(C) Task: AKI Recovery** | | | | **(D) Task: 90-day post-AKI mortality** | | | |
| **Model** | **AUROC** | **AUPRC** | **F1** | **Model** | **AUROC** | **AUPRC** | **F1** |
| BAT-AKI | 0.6080 (0.5745–0.6397) Reference | 0.7417 (0.7082–0.7765) Reference | 0.7748 (0.7535–0.7944) Reference | BAT-AKI | 0.8017 (0.7708–0.8311) Reference | 0.5063 (0.4422–0.5684) Reference | 0.4955 (0.4508–0.5368) Reference |
| HEART | 0.5805 (0.5463–0.6133) P=.03 | 0.7153 (0.6796–0.7516) P=.02 | 0.7769 (0.7564–0.7966) P=.67 | HEART | 0.7831 (0.7531–0.8124) P=.04 | 0.4524 (0.3937–0.5190) P=.006 | 0.4625 (0.4181–0.5042) P=.01 |
| Transformer | 0.5767 (0.5433–0.6093) P=.047 | 0.7097 (0.6751–0.7471) P=.03 | 0.7760 (0.7555–0.7960) P=.81 | Transformer | 0.7617 (0.7265–0.7947) P=.003 | 0.4397 (0.3816–0.5086) P=.04 | 0.4601 (0.4155–0.5027) P=.03 |
| RETAIN | 0.5288 (0.4946–0.5631) P<.001 | 0.6735 (0.6373–0.7119) P<.001 | 0.7909 (0.7709–0.8095) P=.004 | RETAIN | 0.6504 (0.6150–0.6849) P<.001 | 0.2566 (0.2232–0.3025) P<.001 | 0.3711 (0.3310–0.4105) P<.001 |
| LSTM | 0.5480 (0.5134–0.5818) P=.002 | 0.6880 (0.6526–0.7263) P=.004 | 0.7844 (0.7643–0.8036) P=.09 | LSTM | 0.6961 (0.6619–0.7290) P<.001 | 0.3284 (0.2816–0.3877) P<.001 | 0.3922 (0.3466–0.4354) P<.001 |
| XGBoost | 0.5651 (0.5302–0.5990) P=.02 | 0.7069 (0.6717–0.7436) P=.02 | 0.7912 (0.7715–0.8100) P=.003 | XGBoost | 0.7827 (0.7510-0.8131) P=.06 | 0.4864 (0.4223-0.5523) P=.37 | 0.4827 (0.4345-0.5276) P=.39 |

**Table S3.** Performance of BAT-AKI and baseline models on downstream tasks. Values are point estimates with 95% confidence intervals from bootstrap resamples. *P* values are two-sided paired bootstrap comparisons of each baseline against BAT-AKI within the same task and metric; BAT-AKI is the reference.

| ****Model variant**** | ****Token acc.**** | AUROC | AUPRC | ****F1**** |
| --- | --- | --- | --- | --- |
| BAT-AKI – | 0.6082 | 0.8469 (0.8355–0.8585) Reference | 0.5904 (0.5655–0.6155) Reference | 0.4808 (0.4626–0.4989) Reference |
| – semantic | 0.5857 | 0.8339 (0.8215–0.8459) *P*<.001 | 0.5729 (0.5463–0.5990) *P*=.002 | 0.4856 (0.4661–0.5050) *P*=.43 |
| – time | 0.4525 | 0.8314 (0.8194–0.8441) *P*<.001 | 0.5631 (0.5375–0.5891) *P*<.001 | 0.4674 (0.4492–0.4858) *P*=.04 |
| – type | 0.6038 | 0.8376 (0.8253–0.8494) *P*=.002 | 0.5776 (0.5506–0.6040) *P*=.01 | 0.4746 (0.4548–0.4935) *P*=.27 |
| – hontology | 0.4903 | 0.8402 (0.8281–0.8521) *P*=.047 | 0.5770 (0.5509–0.6030) *P*=.02 | 0.4755 (0.4564–0.4939) *P*=.38 |
| – sontology | 0.3799 | 0.8348 (0.8225–0.8464) *P*<.001 | 0.5621 (0.5355–0.5870) *P*<.001 | 0.4948 (0.4745–0.5137) *P*=.045 |

**Table S4.** Ablation results for the embedding components of BAT-AKI. BAT-AKI− is the model trained without the BAP objective, serving as the baseline for evaluating embedding-only performance. Each ablation variant removes one embedding channel from the full model. Token acc. reports Top-1 masked-token prediction accuracy computed from 1,000 sampled test sequences, and AUROC/AUPRC/F1 reflect downstream AKI onset prediction performance, reported as point estimates with 95% confidence intervals from 5000 bootstrap resamples. *P* values are two-sided paired bootstrap comparisons of each ablation variant against BAT-AKI−; BAT-AKI− is the reference. Hontology refers to the hierarchical-ontology embedding component, and sontology refers to the semantic-ontology embedding component.

| ****Model variant**** | ****Token acc. (fluct.-pred/ fluct.-det)**** | AUROC | AUPRC | ****F1**** |
| --- | --- | --- | --- | --- |
| BAT-AKI | 0.6351****/****0.9178 | 0.8549 (0.8433–0.8658) Reference | 0.6041 (0.5786–0.6292) Reference | 0.5054 (0.4849–0.5239) Reference |
| -BAP | 0.6264/0.7595 | 0.8469 (0.8355–0.8585) *P*=.009 | 0.5904 (0.5655–0.6155) *P*=.007 | 0.4808 (0.4626–0.4989) *P*<.001 |

**Table S5.** Ablation of the BAP objective in BAT-AKI. Token acc. (fluct.-pred / fluct.-det) reports fluctuating-token prediction accuracy and fluctuation-detection accuracy, computed from 1,000 sampled test sequences. AUROC/AUPRC/F1 reflect downstream AKI onset prediction performance, reported as point estimates with 95% confidence intervals from 5000 bootstrap resamples. *P* values are two-sided paired bootstrap comparisons of BAT-AKI− against BAT-AKI; BAT-AKI is the reference.

| **Subgroup** | **Model** | **n (AKI %)** | **AUROC (95% CI)** | **AUPRC (95% CI)** | **F1 (95% CI)** |
| --- | --- | --- | --- | --- | --- |
| White | BAT-AKI | 7995 (12.2%) | 0.8592 (0.8455–0.8720) Reference | 0.6093 (0.5780–0.6378) Reference | 0.5078 (0.4849–0.5295) Reference |
| White | Transformer | 7995 (12.2%) | 0.8316 (0.8169–0.8464) *P*<.001 | 0.5497 (0.5181–0.5808) *P*<.001 | 0.4908 (0.4661–0.5145) *P*=.07 |
| Black | BAT-AKI | 1703 (16.1%) | 0.8084 (0.7781–0.8369) Reference | 0.5691 (0.5109–0.6230) Reference | 0.4840 (0.4409–0.5267) Reference |
| Black | Transformer | 1703 (16.1%) | 0.8048 (0.7749–0.8344) *P*=.73 | 0.5277 (0.4655–0.5873) *P*=.02 | 0.4802 (0.4333–0.5263) *P*=.81 |
| Other | BAT-AKI | 1166 (12.5%) | 0.8929 (0.8646–0.9187) Reference | 0.6458 (0.5648–0.7185) Reference | 0.5309 (0.4688–0.5894) Reference |
| Other | Transformer | 1166 (12.5%) | 0.8654 (0.8304–0.8973) *P*=.04 | 0.6107 (0.5271–0.6883) *P*=.15 | 0.5449 (0.4788–0.6049) *P*=.60 |

**Table S6.** AKI-onset performance of BAT-AKI and the Transformer within race subgroups on the internal temporal test set. n (AKI %) denotes the number of encounters and the corresponding AKI incidence. Values are point estimates with 95% confidence intervals from 5000 bootstrap resamples within each subgroup. *P* values are two-sided paired bootstrap comparisons of the Transformer against BAT-AKI within each subgroup and metric; BAT-AKI is the reference.

| **Subgroup** | **Model** | **n (AKI %)** | **AUROC (95% CI)** | **AUPRC (95% CI)** | **F1 (95% CI)** |
| --- | --- | --- | --- | --- | --- |
| <65 | BAT-AKI | 6096 (11.0%) | 0.8547 (0.8383–0.8702) Reference | 0.5677 (0.5301–0.6046) Reference | 0.4873 (0.4589–0.5159) Reference |
| <65 | Transformer | 6096 (11.0%) | 0.8358 (0.8187–0.8522) *P*<.001 | 0.5124 (0.4735–0.5516) *P*<.001 | 0.4644 (0.4350–0.4944) *P*=.07 |
| 65–79 | BAT-AKI | 3474 (14.5%) | 0.8608 (0.8416–0.8799) Reference | 0.6422 (0.5981–0.6850) Reference | 0.5230 (0.4920–0.5539) Reference |
| 65–79 | Transformer | 3474 (14.5%) | 0.8378 (0.8171–0.8581) *P*<.001 | 0.6003 (0.5564–0.6431) *P*=.002 | 0.5247 (0.4919–0.5582) *P*=.90 |
| ≥80 | BAT-AKI | 1294 (17.2%) | 0.8272 (0.7936–0.8596) Reference | 0.6264 (0.5635–0.6845) Reference | 0.5161 (0.4676–0.5612) Reference |
| ≥80 | Transformer | 1294 (17.2%) | 0.7912 (0.7557–0.8258) *P*=.008 | 0.5587 (0.4912–0.6197) *P*<.001 | 0.5108 (0.4585–0.5600) *P*=.80 |

**Table S7.** AKI-onset performance of BAT-AKI and the Transformer within age subgroups on the internal temporal test set. n (AKI %) denotes the number of encounters and the corresponding AKI incidence. Values are point estimates with 95% confidence intervals from 5000 bootstrap resamples within each subgroup. *P* values are two-sided paired bootstrap comparisons of the Transformer against BAT-AKI within each subgroup and metric; BAT-AKI is the reference.

| **Metric** | **Site A** | | **Site B** | | **Site C** | |
| --- | --- | --- | --- | --- | --- | --- |
|  | **BAT-AKI** | **Transformer** | **BAT-AKI** | **Transformer** | **BAT-AKI** | **Transformer** |
|  | **TASK: AKI Onset** | | | | | |
| AUROC | 0.7989 (0.7885–0.8092) Reference | 0.7587 (0.7479–0.7694) *P*<.001 | 0.7911 (0.7770–0.8052) Reference | 0.7546 (0.7395–0.7692) *P*<.001 | 0.7754 (0.7677–0.7827) Reference | 0.7478 (0.7404–0.7551) *P*<.001 |
| AUPRC | 0.5687 (0.5495–0.5871) Reference | 0.4617 (0.4419–0.4819) *P*<.001 | 0.5267 (0.4984–0.5557) Reference | 0.4205 (0.3925–0.4481) *P*<.001 | 0.4777 (0.4631–0.4924) Reference | 0.4090 (0.3951–0.4227) *P*<.001 |
| F1 | 0.4445 (0.4307–0.4581) Reference | 0.4005 (0.3873–0.4140) *P*<.001 | 0.3918 (0.3753–0.4082) Reference | 0.3758 (0.3596–0.3913) *P*<.001 | 0.4209 (0.4112–0.4304) Reference | 0.3545 (0.3465–0.3627) *P*<.001 |
|  | **Task: AKI Early Reversal** | | | | | |
| AUROC | 0.5756 (0.5525–0.5991) Reference | 0.5333 (0.5097–0.5569) P=.007 | 0.5892 (0.5581–0.6204) Reference | 0.5219 (0.4896–0.5558) P=.001 | 0.5814 (0.5660–0.5971) Reference | 0.5464 (0.5302–0.5626) P<.001 |
| AUPRC | 0.7211 (0.6973–0.7459) Reference | 0.6863 (0.6617–0.7116) P=.006 | 0.7146 (0.6807–0.7500) Reference | 0.6656 (0.6316–0.7024) P=.008 | 0.6919 (0.6742–0.7103) Reference | 0.6641 (0.6462–0.6821) P=.001 |
| F1 | 0.7728 (0.7578–0.7872) Reference | 0.7984 (0.7849–0.8117) P<.001 | 0.7551 (0.7337–0.7753) Reference | 0.7899 (0.7707–0.8084) P<.001 | 0.7671 (0.7568–0.7772) Reference | 0.7716 (0.7618–0.7814) P=.007 |
|  | **Task: AKI Recovery** | | | | | |
| AUROC | 0.5903 (0.5661–0.6148) Reference | 0.5375 (0.5119–0.5624) P=.001 | 0.5784 (0.5450–0.6119) Reference | 0.5231 (0.4906–0.5575) P=.009 | 0.5985 (0.5815–0.6160) Reference | 0.5538 (0.5362–0.5712) P<.001 |
| AUPRC | 0.7250 (0.6980–0.7521) Reference | 0.6859 (0.6586–0.7131) P=.003 | 0.7191 (0.6825–0.7567) Reference | 0.6780 (0.6413–0.7160) P=.01 | 0.7219 (0.7031–0.7418) Reference | 0.6913 (0.6718–0.7111) P<.001 |
| F1 | 0.7917 (0.7768–0.8054) Reference | 0.7947 (0.7799–0.8083) P=.02 | 0.7866 (0.7660–0.8060) Reference | 0.7666 (0.7441–0.7880) P=.001 | 0.7833 (0.7726–0.7936) Reference | 0.7887 (0.7785–0.7987) P=.02 |
|  | **Task:** **90-day post-AKI mortality** | | | | | |
| AUROC | 0.7884 (0.7662–0.8099) Reference | 0.7270 (0.7011–0.7509) *P*<.001 | 0.8249 (0.7986–0.8502) Reference | 0.7508 (0.7175–0.7825) *P*<.001 | 0.7308 (0.7156–0.7455) Reference | 0.6742 (0.6573–0.6904) *P*<.001 |
| AUPRC | 0.4471 (0.4006–0.4965) Reference | 0.3200 (0.2841–0.3593) *P*<.001 | 0.5159 (0.4540–0.5802) Reference | 0.4150 (0.3588–0.4772) *P*<.001 | 0.4500 (0.4217–0.4778) Reference | 0.3727 (0.3486–0.3984) *P*<.001 |
| F1 | 0.4684 (0.4350–0.4997) Reference | 0.4162 (0.3850–0.4449) *P*<.001 | 0.5312 (0.4883–0.5714) Reference | 0.4696 (0.4224–0.5136) *P*=.001 | 0.4844 (0.4615–0.5063) Reference | 0.4643 (0.4446–0.4833) *P*=.046 |

**Table S8.** Comparison of model generalizability of BAT-AKI and the Transformer on downstream AKI prediction tasks across external sites. Values are point estimates with 95% confidence intervals from 5000 bootstrap resamples. *P* values are two-sided paired bootstrap comparisons of the Transformer against BAT-AKI within each site, task, and metric; BAT-AKI is the reference.

| **Metric** | **Random equal-size deletion** | **MDRD-excluded** |
| --- | --- | --- |
| AUROC | 0.8415 (0.8266–0.8561) Reference | 0.8320 (0.8165–0.8466) *P*=.38 |
| AUPRC | 0.5721 (0.5383–0.6051) Reference | 0.5504 (0.5188–0.5830) *P*=.36 |
| F1 | 0.5009 (0.4751–0.5267) Reference | 0.4928 (0.4680–0.5171) *P*=.64 |

**Table S9.** AKI-onset performance of BAT-AKI after excluding encounters whose baseline serum creatinine required MDRD back-calculation, compared with a control cohort from which an equal number of randomly selected encounters were removed. Values are point estimates with 95% confidence intervals from 5000 bootstrap resamples. *P* values are two-sided independent bootstrap comparisons based on the bootstrap distribution of the difference between the MDRD-excluded run and the random equal-size deletion control; the random-deletion control is the reference.

| **Category** | **No. concepts** | **Retained concepts** | **Meaning** |
| --- | --- | --- | --- |
| Laboratory measurements | 51 | LAB::LG32892-8; LAB::LG6373-7; LAB::32623-1; LAB::LG49936-4; LAB::LG10990-6; LAB::787-2; LAB::LG13614-9; LAB::LG7967-5; LAB::LG2807-8; LAB::LG11363-5; LAB::LG49864-8; LAB::LG7247-2; LAB::LG32857-1; LAB::LG44868-4; LAB::786-4; LAB::4544-3; LAB::LG32850-6; LAB::48643-1; LAB::LG49881-2; LAB::785-6; LAB::48642-3; LAB::788-0; LAB::LG49763-2; LAB::LG50024-5; LAB::LG6657-3; LAB::LG1314-6; LAB::2703-7; LAB::51733-4; LAB::LG345-5; LAB::LG32886-0; LAB::LG32885-2; LAB::LG32848-0; LAB::LG32849-8; LAB::706-2; LAB::770-8; LAB::713-8; LAB::LG32863-9; LAB::736-9; LAB::5905-5; LAB::LG50041-9; LAB::LG5903-2; LAB::LG49848-1; LAB::LG6199-6; LABC_57735-3; LAB::LG5665-7; LAB::LG49949-7; LAB::LG6426-3; LAB::LG6033-7; LAB::LG1777-4; LAB::LG49422-5; LAB::61151-7 | Platelet count; Chloride; Mean platelet volume; Potassium; Potassium (measurement 2); Mean corpuscular volume; Anion gap; Glucose; Bicarbonate; Sodium; Calcium; Calcium (measurement 2); Leukocyte count; Hemoglobin concentration; Mean corpuscular hemoglobin concentration; Hematocrit; Erythrocyte count; eGFR (MDRD, Black); Glucose (measurement 2); Mean corpuscular hemoglobin; eGFR (MDRD, non-Black); Red blood cell distribution width; Blood urea nitrogen; Creatinine; Creatinine (measurement 2); Blood urea nitrogen (measurement 2); Arterial oxygen partial pressure; Oxygen saturation; Blood pH; Neutrophil count; Monocyte count; Basophil count; Eosinophil count; Basophil percentage; Neutrophil percentage; Eosinophil percentage; Lymphocyte count; Lymphocyte percentage; Monocyte percentage; Magnesium; Magnesium (measurement 2); Total bilirubin; Total bilirubin (measurement 2); Urine protein qualitative result; Alkaline phosphatase; Phosphate; Phosphate (measurement 2); Aspartate aminotransferase; Total protein concentration; Total protein; Albumin |
| Medications | 24 | MED:ATC:B01AB; MED:ATC:B05XA; MED:ATC:N02BE; MED:ATC:B05CB; MED:ATC:A12CA; MED:ATC:C03CA; MED:ATC:A12BA; MED:ATC:N02AA; MED:ATC:A02AA; MED:ATC:G04BX; MED:ATC:A12AA; MED:ATC:G04BA; MED:ATC:J01CG; MED:ATC:J01DB; MED:RX:1013021; MED:ATC:H02AB; MED:ATC:A07AA; MED:ATC:J01XA; MED:ATC:A04AA; MED:RX:1807628; MED:ATC:V08AB; MED:ATC:N01BB; MED:ATC:N01AH; MED:ATC:N02AB | Heparin-group anticoagulants; Intravenous electrolyte solutions; Anilide analgesics/antipyretics; Salt solutions; Sodium supplements; High-ceiling diuretics; Potassium supplements; Natural opium alkaloids; Magnesium antacids; Other urologicals; Calcium supplements; Urinary acidifiers; Beta-lactamase inhibitors; First-generation cephalosporins; Lidocaine; Glucocorticoids; Intestinal antiinfectives; Glycopeptide antibacterials; 5-HT3 antagonists; Sodium chloride 0.9% injection; Low-osmolar water-soluble X-ray contrast media; Amide local anesthetics; Opioid anesthetics; Opioid analgesics |
| Procedures | 30 | PX:CH:36415; PX:CH:99232; PX:CH:97116; PX:CH:J1650; PX:CH:80048; PX:CH:85027; PX:CH:J7030; PX:CH:99233; PX:CH:J7120; PX:CH:J1100; PX:CH:97535; PX:CH:83880; PX:CH:J0690; PX:CH:82803; PX:CH:99231; PX:CH:85025; PX:CH:94760; PX:CH:J2543; PX:CH:97001; PX:CH:99285; PX:CH:83735; PX:CH:J2405; PX:CH:Q9967; PX:CH:99222; PX:CH:J1815; PX:CH:80053; PX:CH:84100; PX:CH:J2250; PX:CH:J3010; PX:CH:99223 | Routine venipuncture; Subsequent hospital care; Gait training; Enoxaparin injection; Basic metabolic panel; Complete blood count; Normal saline infusion; Subsequent hospital care (2); Ringer lactate infusion; Dexamethasone injection; Self-care training; Natriuretic peptide test; Cefazolin injection; Blood gas analysis; Subsequent hospital care (3); Complete blood count with differential; Blood oxygen level measurement; Piperacillin-tazobactam injection; Physical therapy evaluation; Emergency department visit; Magnesium test; Ondansetron injection; Low-osmolar contrast administration; Initial hospital care; Insulin injection; Comprehensive metabolic panel; Phosphorus test; Midazolam injection; Fentanyl injection; Initial hospital care (2) |
| Diagnoses | 12 | DX:09:585; DX:09:584; DX:09:428; DX:09:250; DX:09:429; DX:09:401; DX:09:285; DX:09:414; DX:09:V58; DX:09:518; DX:09:786; DX:09:276 | Chronic kidney disease; Acute kidney failure; Heart failure; Diabetes mellitus; Other heart disease; Essential hypertension; Anemia; Coronary atherosclerosis; Aftercare and long-term therapy encounters; Respiratory failure and related pulmonary conditions; Respiratory symptoms; Electrolyte/fluid disorders |
| Vital signs | 2 | SYSTOLIC; DIASTOLIC | Systolic blood pressure; Diastolic blood pressure |

**Table S10.** Retained clinical concepts in the AKI-related lexicon (*V_aki_*). Concepts appearing more than once are numbered, indicating that multiple distinct codes for the same clinical concept were retained. Two medication concepts without a valid ATC mapping are listed under their original RxNorm identifiers.

| **Data split** | **Total encounters, N** | **Encounters containing DX:09:584, n (%)** |
| --- | --- | --- |
| Training | 33,115 | 4,213 (12.72) |
| Validation | 4,794 | 631 (13.16) |
| Internal temporal test | 10,864 | 1,766 (16.26) |

**Table S11.** Frequency of the preadmission acute kidney failure diagnosis DX:09:584 in the AKI-onset data splits. Encounters were counted once if the diagnosis appeared at least once.

| **Input specification** | **AUROC (95% CI)** | **AUPRC (95% CI)** | **F1 (95% CI)** |
| --- | --- | --- | --- |
| Original BAT-AKI | 0.8549 (0.8434–0.8661) | 0.6041 (0.5785–0.6295) | 0.5054 (0.4859–0.5243) |
| DX:09:584 removed | 0.8561 (0.8448–0.8674) | 0.6033 (0.5778–0.6287) | 0.5125 (0.4935–0.5312) |
| *P* value | .462 | .822 | .132 |

**Table S12.** AKI-onset performance of BAT-AKI after removing DX:09:584 from the input sequences. Values are point estimates with 95% confidence intervals from 5000 bootstrap resamples. *P* values are two-sided paired bootstrap comparisons of the code-removed model against the original BAT-AKI model; the original model is the reference.

| **Task** | **Weighting strategy** | **AUROC (95% CI)** | **AUPRC (95% CI)** | **F1 (95% CI)** |
| --- | --- | --- | --- | --- |
| AKI early reversal | Equal weighting | 0.6006 (0.5703–0.6318) | 0.7036 (0.6690–0.7388) | 0.7596 (0.7394–0.7790) |
|  | Class weighting | 0.5961 (0.5644–0.6251) | 0.6956 (0.6617–0.7304) | 0.6248 (0.5960–0.6514) |
| AKI recovery | Equal weighting | 0.6080 (0.5745–0.6397) | 0.7417 (0.7082–0.7765) | 0.7748 (0.7535–0.7944) |
|  | Class weighting | 0.6115 (0.5784–0.6432) | 0.7468 (0.7130–0.7805) | 0.6426 (0.6135–0.6707) |

**Table S13.** Comparison of weighting strategies for BAT-AKI on AKI early reversal and recovery. Class weighting indicates $w_{\mathrm{pos}}={N_{\mathrm{neg}}}/{N_{\mathrm{pos}}}$, whereas equal weighting indicates $w_{\mathrm{pos}}=1$. Values are point estimates with 95% confidence intervals from 5000 encounter-level bootstrap resamples. F1-scores were calculated using a fixed probability threshold of 0.5.

**Figure S1.** Comparison of BAP strategies under different perturbation ratios

**Figure S2.** Symmetric BAP strategy under different MLM masking ratios and BAP perturbation ratios.

 **Figure S3.** This figure presents the complete color mapping of all tokens shown in Figure 6A, which visualizes the fluctuation trajectories of three key laboratory measurements. Each color uniquely corresponds to a clinical token (diagnosis, medication, laboratory test, or procedure) that appeared among the top-10 attention sources.

**Figure S4.** Concept importance for the case study (the same encounter as Figure 6; sample index 2648). Concept-level IG attributions for the AKI-onset logit were ranked by absolute value, with the top 20 concepts shown. Bars reflect magnitude only; the direction of contribution is not shown.

**Figure S5.** Cohort-level concept importance for AKI-onset prediction by BAT-AKI. Integrated-gradients (IG) attributions were computed per token, collapsed to clinical concepts within each encounter, and summarized as the mean absolute attribution across the AKI-onset test set. Bars reflect magnitude only; the direction of contribution is not shown.

**Figure S6.** Calibration curves compare predicted AKI onset probabilities with observed event rates on the held-out internal evaluation subset. Raw model probabilities and Platt-calibrated probabilities are shown for BAT-AKI and benchmark models. The dashed diagonal line indicates perfect calibration. Platt scaling was fitted on a fixed stratified calibration subset and evaluated on a separate held-out evaluation subset. Brier scores (raw / Platt-calibrated; lower is better) were: BAT-AKI 0.1357 / 0.0779; HEART 0.1384 / 0.0834; RETAIN 0.1435 / 0.0828; XGBoost 0.1522 / 0.0827; Transformer 0.1534 / 0.0824; LSTM 0.1839 / 0.0893.

**Figure S7.** Decision curves show the net benefit of BAT-AKI and benchmark models across threshold probabilities for AKI onset prediction on the held-out internal evaluation subset. Raw and Platt-calibrated probabilities are shown together with treat-all and treat-none reference strategies.

**References**

1.	Khwaja A. KDIGO clinical practice guidelines for acute kidney injury. Nephron Clin Pract. 2012;120(4):c179-c184. doi:10.1159/000339789

2.	Bouchard J. Estimating Baseline Serum Creatinine for Assessing Acute Kidney Injury: Not a One Size Fits All Approach. Kidney Int Rep. 2021;6(3):562-564. doi:10.1016/j.ekir.2021.01.030

3.	Cama-Olivares A, Braun C, Takeuchi T, et al. Systematic Review and Meta-Analysis of Machine Learning Models for Acute Kidney Injury Risk Classification. J Am Soc Nephrol. 2025;36(10):1969-1983. doi:10.1681/ASN.0000000702

4.	Feng Y, Wang AY, Jun M, et al. Characterization of Risk Prediction Models for Acute Kidney Injury: A Systematic Review and Meta-analysis. JAMA Netw Open. 2023;6(5):e2313359. doi:10.1001/jamanetworkopen.2023.13359

5.	Cartin-Ceba R, Kashiouris M, Plataki M, Kor DJ, Gajic O, Casey ET. Risk factors for development of acute kidney injury in critically ill patients: a systematic review and meta-analysis of observational studies. Crit Care Res Pract. 2012;2012:691013. doi:10.1155/2012/691013

6.	Huang T, Rizvi SA, Thakur RK, et al. HEART: Learning better representation of EHR data with a heterogeneous relation-aware transformer. J Biomed Inform. 2024;159:104741. doi:10.1016/j.jbi.2024.104741

7.	Sundararajan M, Taly A, Yan Q. Axiomatic Attribution for Deep Networks. In: Proceedings of the 34th International Conference on Machine Learning (ICML). PMLR; 2017:3319-3328.
